# Microbial communities with distinct denitrification potential in spruce and beech soils differing in nitrate leaching

**DOI:** 10.1038/s41598-017-08554-1

**Published:** 2017-08-29

**Authors:** Jiří Bárta, Karolina Tahovská, Hana Šantrůčková, Filip Oulehle

**Affiliations:** 1 Department of Ecosystem Biology, Faculty of Science, University of South Bohemia, Branišovská 31, 370 05 České Budějovice, Czech Republic; 2 Czech Geological Survey, Department of Environmental Geochemistry and Biogeochemistry, Prague, 118 21 Czech Republic

## Abstract

Nitrogen leaching owing to elevated acid deposition remains the main ecosystem threat worldwide. We aimed to contribute to the understanding of the highly variable nitrate losses observed in Europe after acid deposition retreat. Our study proceeded in adjacent beech and spruce forests undergoing acidification recovery and differing in nitrate leaching. We reconstructed soil microbial functional characteristics connected with nitrogen and carbon cycling based on community composition. Our results showed that in the more acidic spruce soil with high carbon content, where Acidobacteria and Actinobacteria were abundant (Proteo:Acido = 1.3), the potential for nitrate reduction and loss via denitrification was high (denitrification: dissimilative nitrogen reduction to ammonium (DNRA) = 3). In the less acidic beech stand with low carbon content, but high nitrogen availability, Proteobacteria were more abundant (Proteo:Acido = 1.6). Proportionally less nitrate could be denitrified there (denitrification:DNRA = 1), possibly increasing its availability. Among 10 potential keystone species, microbes capable of DNRA were identified in the beech soil while instead denitrifiers dominated in the spruce soil. In spite of the former acid deposition impact, distinct microbial functional guilds developed under different vegetational dominance, resulting in different N immobilization potentials, possibly influencing the ecosystem’s nitrogen retention ability.

## Introduction

Since the industrial period, atmospheric sulphur (S) and nitrogen (N) deposition has become one of the main drivers for changing ecosystem biogeochemistry. The main consequences of long-term S and N loading lie in soil acidification and the interlinked changes in plant productivity and diversity^1–3^, soil carbon and nutrient cycling^4^ and alteration in the soil microbial community structure^5^. Besides soil acidification, long-term N deposition can lead to an ecosystem’s N saturation where the excess N may be lost in the form of nitrates^6^. Reduced depositions in the last decades started the recovery of many European ecosystems, accompanied only in some of them by reduced nitrate leaching^7,8^.

Apart from plants, soil microbes, as essential mediators of all assimilative and dissimilative N transformation processes, play a key role in the soil mineral N balance. Nitrates accumulate in soil either under high nitrification rates and/or low nitrate reduction rates (i.e. low microbial immobilization, denitrification or dissimilative nitrate reduction to ammonium (DNRA)). Generally, it is the heterotrophic community (usually prevailing over the autotrophic), being dependent on soil carbon (C), which regulates whether N is lost or retained in the soil^9–11^. Although we have now advanced ability to explore structures of soil microbial communities, there is still a need for studies focusing on specific links between microbial taxonomic and functional diversity and their participation in soil C and N transformation and eventually soil N retention^12^.

The effect of elevated N input on soil microbial communities has been widely discussed with most studies drawing the conclusion of decreasing fungal biomass and activity, particularly mycorrhizas^5,13–15^. Lower fungal biomass and thus lower activity of the lignin-degrading enzymes^16,17^ shift microbial utilization to easily available C and after these are quickly exhausted it may lead to overall C limitation of the microbial community^4,18–21^. As a consequence, N mineralization and nitrate concentrations increase^22–25^.

Elevated N loading and subsequent changes in microbial utilization of organic C can change the overall structure of a soil prokaryotic community. Particularly, copiotrophic taxa (r-strategists) namely Alpha- and Gammaproteobacteria increase at elevated N input^26–28^. In contrast, Acidobacteria, a group which is mostly considered as oligotrophic (K-strategists), decline with increasing N loads^27,29^. Functional metagenomic analyses showed higher relative abundances of specific gene categories associated with DNA/RNA replication, electron transport and protein metabolism after N amendments. This indicates higher growth and metabolic activity typical for copiotrophs^27^. Such community shifts may lead to changes in substrate use efficiencies since copiotrophs are supposed to grow faster but with lower growth efficiency^30^.

The Czech side of the central European area of the so-called “Black Triangle” located along the German-Polish-Czech border and belongs among the regions most affected by acid pollution^31^. Since the 1980’s, a considerable decline in S and N deposition has occurred (more than 90% and 40% reduction, respectively) due to the restructuring of industrial and agricultural practices^31^. Here we investigated the microbial community structure using DNA sequencing in the beech and spruce soils of two adjacent forests and currently differing in their nitrate leaching^7^. We combined molecular identification data with biogeochemical soil and microbial characteristics to explore the links among microbial community composition and N transformation processes. Our primary question was whether variations in microbial community structure could help to explain observed differences in nitrate leaching between both forests. We supposed that the microbial community structure could be different between both forests due to different vegetation type per se (i.e. differences in litter composition and input, different levels of dry deposition etc.). We hypothesized that due to historically high acid deposition, the fungal abundances would be rather similar probably with a shift to saprotrophic strategy. Furthermore, the bacterial community would be dominated by Acidobacteria in both forests due to very low soil pH. However, we also recognized that copiotrophic taxa might be favoured in the beech soils that could correspond to richer N conditions there.

## Materials and Methods

### Sampling sites

Our experimental site, Načetín, is located on the ridge of the Ore Mountains, in the north-western part of the Czech Republic (Fig. S1). This region was exposed to extremely high acid deposition in the past^32^ and has currently been undergoing recovery since the 1990s^31^ (Fig. S3). The site has been intensively used for the monitoring of atmospheric deposition, soil and soil water chemistry since 1993. Two adjacent stands were studied. The spruce stand (50°35′26″N, 13°15′14″E) is located at an elevation of 784 m a.s.l. on a gentle slope oriented to the northwest and is completely dominated by Norway spruce (*Picea abies*, ~80 years old) with the understory vegetation dominated by wavy hair-grass (*Deschampsia flexuosa*), bushgrass and blueberry (Fig. S2). The beech stand (50°35′22″N, 13°16′07″E) lies at an elevation of 823 m a.s.l. and is composed of European beech (*Fagus sylvatica*, ~120 years old, spruce trees continuously died off due to the air pollution) with no understory vegetation present (only the limited presence of beech seedlings). The bedrock of both stands is gneiss, and soils are dominated by dystric cambisols. The snow cover lasts usually from November to April; the average annual temperature is 6.3 °C, and annual precipitation is ~1000 mm. Bulk N deposition averaged 11 kg N ha^−1^ year^−1^ and throughfall N flux was similar among stands 16 kg ha^−1^ year^−1^ ^33^. In total, 16 real composite soil samples (based on two cores) were taken from 16 randomly selected plots (9 m^2^) in each of both forests in early May 2013. We sampled the upper organic soil layer (Of + Oh + A horizons), using a soil corer (up to 10–15 cm).

### Soil solution sampling and analysis

Soil pore water samples from the forest floor were collected using Rhizon® suction samplers (Rhizosphere Research Products, NL), comprising 10 cm long, 2.5 mm diameter porous membranes attached to 50 ml syringes. In each forest, four to six suction lysimeters were placed in 16 randomly selected plots (9 m^2^). Samples were collected by applying suction overnight, and a composite sample was made in the morning to get one representative sample per plot (n = 16). The sampling was performed twice in May 2013 in all 16 plots and the averages are used. Samples were stored at 4 °C and analysed immediately after arrival in laboratory. Sulphates and nitrates were measured by ion exchange chromatography (Alltech 650, USA). Base cations and total aluminium were determined by flame atomic absorption spectrometry (AAnalyst 200 Perkin-Elmer, USA). Ammonium was determined by indophenol blue colorimetry. Dissolved organic carbon (DOC) was measured by a nondispersive infrared detector after combustion to CO_2_. Dissolved nitrogen (DN) was determined after sample combustion to NO and its reaction with O_3_. For details see Oulehle *et al*.^33^.

### Biochemical soil properties and microbial activity

Soil pH was determined in deionized water (water:soil, 25:10, v/w) by agitating for 5 min and letting stand for 0.5 h. Dried (60 °C) and finely ground soil samples were analysed for total C (Ctot) and N (Ntot) content on an NC elemental analyser (Vario micro cube, Elementar Analysensysteme GmbH, Germany). The molar C to N ratio of the soil was calculated. Moist soil samples (10 g, 60% of water holding capacity) were placed in glass flasks sealed with perforated Parafilm and incubated for one or three weeks (10 °C). After one week incubation, the soil was either extracted (0.5 M K_2_SO_4_, extractant: soil, 4: 1, v/w, agitated for 45 min) or fumigated for one day with chloroform (amylene stabilized) before extraction. Then, extracts were centrifuged (4000 g, 10 min) and filtered through 0.45 µm glass fibre filter. Non-fumigated extracts were analysed for N-NH_4_ and N-NO_3_ using the Flow injection analyser (FIA, QuickChem 8500, Lachat Instruments, USA). Together with fumigated extracts, they were analysed for total carbon (DOC) and nitrogen (TN) content on an elemental analyser (LiqiTOC II, Elementar Analysensysteme GmbH, Germany). Microbial C (Cmic) and N (Nmic) were calculated as the difference in sulphate extractable carbon and nitrogen, respectively, between the fumigated and non-fumigated samples and corrected by the extraction efficiency factors of 0.38 for microbial C^34^, and 0.54 for microbial N (K_*EN*_)^35^. The remaining samples were after three weeks extracted in the same manner and N-NH_4_ and N-NO_3_ were analysed to determine net N mineralization and nitrification rate^36^. Net N mineralization and nitrification rates were calculated as the difference between the final (21 days) and initial (7 days) concentrations of NH_4_^+^ and NO_3_^−^, respectively, divided by the number of days^37^. Carbon use efficiency was calculated from available substrate (soil leachate) and microbial C/N ratios^38^. Nitrogen use efficiency was calculated as a multiple of CUE * available substrate C/N ratio (soil leachate) divided by C/N ratio of microbial biomass^39^. All measurements were performed in two laboratory replications for each soil sample. All data were expressed on a soil dry weight basis (105 °C). Microbial respiration was measured as CO_2_ production after one week’s incubation of soil in hermetically closed vials in the dark (10 g, 60% of water holding capacity, 10 °C), using gas chromatography (Agilent GC HP 6850, USA). The specific respiration rate was calculated as a ratio of respiration rate and Cmic.

### Extracellular enzyme activities

Extracellular enzyme activities were determined by microplate fluorometric assays under standard conditions. For determination of all hydrolytic enzyme activities, 1 g soil was suspended in 100 ml of distilled water and sonicated for 4 min to disrupt soil particles. 200 µL soil suspension was then added to 50 µL methylumbelliferyl (MUF) of substrate solution for β-glucosidase, exocellulase (cellobiohydrolase), phosphatase and N-acetyl-glucosaminidase determination or to 50 µL of 7-aminomethyl-4-coumarin (AMC) substrate solution for leucine-aminopeptidase determination^40^. Three concentrations of each fluorogenic substrate were tested (50, 100 and 300 μM) and the one with the highest enzymatic activity where the enzyme is saturated was picked. Plates were incubated at 20 °C for 120 min. Fluorescence was quantified at an excitation wavelength 365 nm and emission wavelength 450 nm using Infinite F200 microplate reader (TECAN, Germany).

### DNA extraction

Approximately 0.5 g of soil was added to a FastPrep^TM^ Lysis Matrix E tube (MP Biomedicals, Solon, OH, USA). Hexadecyltrimethylammonium bromide (CTAB) extraction buffer, containing 5% CTAB (in 0.7 M NaCl, 120 mM potassium phosphate, pH 8.0) and 0.5 ml phenol-chloroform-isoamylalcohol (25:24:1), was added and agitated in a FastPrep Instrument (MP Biomedicals, Solon, OH, USA) at speed 5–6 for 45 s. After bead beating, the samples were extracted with chloroform and precipitated in a PEG 6000/1.6 M NaCl solution. Pellets were washed with 70% ethanol and re-suspended in molecular biology grade water. Total DNA was quantified using known concentration of genomic DNA which was used for creation of calibration curve and after addition of fluorescent dye SybrGreen the fluorescent signal was compared with unknown samples^41^.

### Quantification of prokaryotic and eukaryotic microbial community

Quantification of bacterial, archaeal and fungal SSU rRNA genes was performed using the FastStart SybrGREEN Roche^®^ Supermix and Step One system (Life Technologies, USA). Each reaction mixture (20 µl) contained 2 µl DNA template (~1–2 ng DNA), 1 µl each primer (0.5 pmol µl^−1^ each, final concentration), 6 µl dH_2_O, 10 µl FastStart SybrGREEN Roche^®^ Supermix (Roche, France) and 1 µl BSA (Fermentas, 20 mg µl^−1^). Initial denaturation (3 min, 95 °C) was followed by 30 cycles of 30 s at 95 °C, 30 s at 62 °C (bacteria) and 60 °C (archaea), 15 s at 72 °C, and completed by fluorescence data acquisition at 80 °C used for target quantification. Product specificity was confirmed by melting point analysis (52 °C to 95 °C with a plate read every 0.5 °C) and amplicon size was verified with agarose gel electrophoresis. Bacterial and archaeal DNA standards consisted of a dilution series (ranging from 10^1^ to 10^9^ gene copies µl^−1^) of a known amount of purified PCR product obtained from genomic *Escherichia coli ATCC 9637* and *Pyrococcus furiosus DSM 3639* DNA by using the SSU gene-specific primers 341F/534R and ARC78F/ARC1059R, respectively^42,43^. R^2^ values for the standard curves were >0.99. Slope values were −>3.37 giving an estimated amplification efficiency of >93%.

The qPCR conditions for fungal quantification were as follows: initial denaturation (10 min, 95 °C) followed by 40 cycles of 1 min at 95 °C, 1 min at 56 °C, 1 min at 72 °C, and completed by fluorescence data acquisition at 72 °C used for target quantification. Fungal DNA standards consisted of a dilution series (ranging from 10^1^ to 10^7^ gene copies µl^−1^) of a known amount of purified PCR product obtained from genomic *Aspergillus niger* DNA by using the SSU gene-specific primers nu-SSU-0817-5′ and nu-SSU1196-3′^44^. R^2^ values for the fungal standard curves were >0.99. The slope was between −3.34 to −3.53 giving estimated amplification efficiency between 95 and 93%, respectively.

Detection limits for the various assays (i.e. lowest standard concentration that is significantly different from the non-template controls) were less than 100 gene copies for each of the genes per assay. Samples, standards and non-template controls were run in duplicates. To deal with potential inhibition during PCR the enhancers (BSA, DMSO) were added to the PCR mixture. Also several dilutions (10x, 20x, 50x, 100x, 1000x) for each sample were tested to see the dilution effect on Ct values.

### Analyses of prokaryotic and fungal community composition

The aliquots of DNA extracts were sent to ARGONE Lab (Illinois, USA) for the preparation of a library and sequencing using MiSeq platform. The Earth Microbiome Project (EMP) protocol was used for library preparation with modified universal primers 515FB/806RB^45^ and ITS1F/ITS2^46^ for prokaryotic 16S rDNA and fungal ITS1 amplicons, respectively. The coverage of prokaryotic primer pair 515FB/806RB was additionally tested in-silico using ARB Silva database release 128. The primer pair 515FB/806RB covers almost uniformly all major bacterial and archaeal phyla (Table S3). Both bacterial 16SrDNA and fungal ITS1 raw pair-end reads (150 bp) were joined using ea-utils to obtain reads of approx. 250 bp length^47^. Quality filtering of reads was applied as previously described^45^. After quality filtering the sequences were trimmed to 250 bp. We obtained 576,133 bacterial and 806,387 fungal sequences after joining and quality trimming. Before picking the operational taxonomic units (OTU), the fungal ITS1 region was extracted from reads using ITSx algorithm^48^. Both 16S and ITS1 amplicons were trimmed to equal lengths in order to avoid spurious OTU clusters^49^. Bacterial reads were clustered (more than 97% similarity) to OTUs using an open-reference OTU picking protocol (QIIME 1.9.1^47^, first with uclust^49^ being applied to search sequences against a Greengenes version 13_05 and Silva 119 database^50^. Taxonomy was assigned to each read by accepting the Greengenes or Silva119 taxonomy string of the best matching Greengenes or Silva119 sequence. Fungal reads were clustered to OTUs using open-reference OTU picking protocol (sequence similarity 98.5%) using UNITE ver. 5.3.2015 database^51^. Blast algorithm (e-value ≤ 0.001) was used for taxonomic assignment. FUNguild algorithm^52^ was then used for the life style fungal assignments.

Alpha diversity metrics, Shannon diversity, Chao1 richness, and Faith’s phylogenetic diversity were calculated after rarefying all samples to the same sequencing depth of 10,000 and 4,500 sequences for prokaryota and fungi, respectively. Prior to computing the Unifrac distances, singleton OTUs (i.e. OTUs with only one sequence) were filtered out, as these are likely to represent sequencing or PCR errors and/or chimeras.

Raw sequences of 16SrDNA and ITS1 amplicons were deposited in European Nucleotide Archive (ENA) under study ID PRJEB17634.

### Network analyses and determination of keystone species

In microbial network analyses it is recommended to use absolute^53^ instead of relative OTU abundances, since relative abundances can create false correlations between OTUs. Therefore, we recalculated relative abundances in each sample of the bacterial and archaeal OTU table to absolute abundances, using the data of bacterial and archaeal SSU gene copies per ng of DNA. This gave us absolute abundances of each OTU in the OTU table. We performed the recommended calculations (n_eff_, sparsity)^54^ regarding the composition of prokaryotic and fungal filtered OTU tables (i.e. removing very rare OTUs by sorting the average OTU abundances in samples from maximum to minimum and choosing only those OTUs which were presented in at least 10 samples out of 31 for prokaryotic community, and for fungal community 5 out of 22. Based on the sparsity of filtered OTU tables we chose the CoNet network algorithm as the relevant calculation method. Unstable edges were filtered out on alpha level of 0.05. We additionally increase the alpha level to 0.01 and rerun the network analyses to confirm the robustness of the network composition (Fig. S14). The resulting OTU tables, separately for beech (15 samples for prokaryota, 11 samples for fungi) and spruce (16 samples for prokaryota, 11 samples for fungi) were used for microbial network analyses. The analysis was done in Cytoscape 3.0.2 with the CoNET application^55,56^. The parameters and settings for network analyses in CoNET application were: -parent_child_exclusion, -row_minocc 8, -correlations (Spearman, Pearson, mutual information, Bray Curtis dissimilatory and Kullback-Leibler dissimilatory). The threshold for edge selection was set to 1,000 top and bottom. During randomization, 100 iterations were calculated for edge scores. In the following bootstrap step 100 iterations were calculated and unstable edges were filtered out (p-level threshold of 0.05). We additionally increase the p-level threshold to 0.01 and rerun the network analyses (Fig. S14). The Brown method was chosen as the p-value merging method and the Benjaminihochberg method for multiple test correction. The resulting network for the spruce and beech prokaryotic community was visualized and analyzed (i.e. degree of nodes, betweenness centrality, closeness centrality) in Cytoscape 3.0.2 and potential keystone OTUs in the beech and spruce forests were identified^53^.

### Analyses of metabolic potential of the prokaryotic community

Two independent pipelines (PICRUSt and RDP FunGene) were used for in-silico prediction of the functional potential of the microbial community. For PICRUSt^57^ analyses the rarified OTU tables to 3,900 sequences generated by Qiime 1.9.1 were used with taxonomic classification based on GreenGenes database ver. 13.05 using closed reference OUT picking method. In general, the PICRUSt pipeline first normalized each OTU abundances by SSU copy variation in bacterial genomes based on the most similar taxa. The resulting normalized table was then used for OTU functional annotation using known bacterial and archaeal genomes^57^. To validate accuracy of PICRUSt metagenomics prediction the Nearest Sequenced Taxon Index (NSTI score) was calculated. The NSTI score was 0.13 ± 0.01 and 0.14 ± 0.01 for beech and spruce, respectively. According to ref.^57^ these NTSI scores are typical for soil samples (NTSI approx. 0.17).

The lists of bacterial and archaeal species for each individual gene was downloaded from the FunGene database^58^. Genes below without specific annotation to the gene family were manually excluded from the analyses. The condensed list of unique genera was created and used for functional annotation in an OTU table (Table S2). By combining SSU qPCR data and relative abundances of assigned bacterial genera, we were able to quantitatively identify the main N transformation pathways.

### Statistics

Differences between the beech and spruce stands were examined for the biochemical soil and microbial parameters using one way ANOVA (Statistica 9.1, Statsoft, Inc.). The normality of distributions and homogeneity of variances were checked using histogram plots and Hartley-Cochran-Bartlett’s tests. Correlations between selected characteristics were explored to find the most important relations.

Principal component analysis (PCA) was employed to compare microbial community composition (relative abundances) of both forests using Canoco 5.0^59^. Then a constrained ordination redundancy analysis (RDA) was used to evaluate the relations between all measured biochemical parameters of soil (log transformed, except pH, explanatory variables) and the microbial community composition (log transformed relative abundance of each phyla, response variables). The contribution of each explanatory variable was tested by forward selection and the Monte-Carlo permutation test (p < 0.05). Only those explanatory variables that showed significant effects were included in the diagrams. The length of the gradient was tested by detrended correspondence analysis prior the selection of the appropriate ordination method. Metric (MDS) and nonmetric distance analyses (NMDS) were done on rarefied OTU tables in R-studio (R 3.4.0) using phyloseq package^60^.

## Results

### Soil characteristics and microbial activity

The soils in both stands were highly acidic with pH ranging from 3.98 to 4.57 and from 3.92 to 4.33 in the beech and spruce soils, respectively (Table 1). Lower pH in the spruce soil was connected with lower base cations (Ca^2+^ and Mg^2+^), and higher aluminium, and sulphate content in the soil leachate.

**Table 1:** Chemical properties of soil and soil solution (n = 16) and spruce (n = 16) soils.

	Beech	Spruce	stat.
pH_H2O_	3.98–4.57	3.92–4.33	**
Calcium (Ca^2+^, µmol.L^−1^)	15.2 (8.7)	9.0 (4.0)	*
Magnesium (Mg^2+^, µmol.L^−1^)	25.9 (10.3)	14.8 (4.9)	***
Sulphates (SO_4_^2−^, µmol.L^−1^)	65.5 (28.3)	85.4 (36.7)	*
Total aluminium (Al_TOT_, µmol.L^−1^)	23.0 (11.1)	36.3 (12.2)	**
Total carbon (C_TOT_, mmol.g^−1^)	30.3 (4.8)	33.5 (1.3)	**
Total nitrogen (N_TOT_, mmol.g^−1^)	1.3 (0.2)	1.3 (0.1)	ns
C_TOT_/N_TOT_ (molar)	23.0 (1.0)	26.3 (1.7)	***
Ammonia (NH_4_^+^, µmol.L^−1^)	2.8 (2.7)	1.0 (0.6)	ns
Nitrates (NO_3_^−^, µmol.L^−1^)	38.2 (48.9)	4.5 (5.7)	**
Dissolved N (DN, µmol.L^−1^)	66.9 (61.2)	25.2 (10.2)	*
Dissolved C (DOC, µmol.L^−1^)	512 (242)	764 (378)	*
DOC/DN	14.0 (9.0)	30.7 (8.9)	***
DOC/NO_3_^−^	35.8 (39.5)	290.0 (217.8)	ns

Statistical significances are marked by asterisks as follows: *p < 0.05, **p < 0.01, ***p < 0.001, ns – not significant.

The spruce soils contained more soil carbon (C_TOT_) than the beech soils, but had the same amount of nitrogen (N_TOT_), resulting in higher spruce total C/N ratio (Table 1). Similarly, the relative proportion of available C to N (DOC/DN) in the spruce soil was roughly double the quantity found (31) in the beech soils (14). The most pronounced difference between both soils was revealed by the proportion of DOC to NO_3_^−^, which was eight times higher (290) in the spruce soil (36), mainly due to very low NO_3_^−^ concentration in the soil solution.

Moreover, the spruce soil displayed a greater rate of net N mineralization (ammonification + nitrification) (Table 2). However, this was mainly due to an elevated net ammonification rate that was twice as high in the spruce when compared with the beech soil. Net nitrification was generally very low in both soils with no difference due to high data variability of both forests. Interestingly, the N mineralization pattern was not in accordance with N availability. There was more mineral N (NH_4_^+^  + NO_3_^−^, Table 1) available in beech soils with eight times higher concentration of NO_3_^−^ in the soil solution; however, N mineralization potential was two times lower.

**Table 2:** Biochemical properties of beech (n = 16) and spruce (n = 16) soils.

	Beech	Spruce	stat.
Microbial carbon (C_mic_, µmol.g^−1^)	346.2 (68.9)	265.1 (77.3)	**
Microbial nitrogen (N_mic_, µmol.g^−1^)	45.5 (11.3)	25.8 (9.0)	***
C_mic_/N_mic_	7.9 (1.5)	11.0 (2.2)	***
C_mic_/C_TOT_	11.1 (2.8)	7.9 (2.3)	***
C_mic_/DOC	6.4 (1.4)	5.1 (1.3)	*
Basal respiration (µmol.g^−1^d^−1^)	3.1 (1.2)	3.1 (0.7)	ns
Specific respiration (nmol C.g C_mic_^−1^.d^−1^)	8.9 (2.7)	12.3 (3.7)	**
Carbon use efficiency (CUE)^a^	0.36 (0.16)	0.24 (0.06)	**
Nitrogen use efficiency (NUE)^a^	0.51 (0.13)	0.66 (0.12)	**
Critical C/N ratio (C:N_CR_)	25.6 (11.5)	46.0 (8.3)	***
Net ammonification (nmol.g^−1^.h^−1^)	130 (80)	250 (140)	**
Net nitrification (nmol.g^−1^.h^−1^)	28 (12)	11(7)	ns
β-Glucosidase (BG, µmol.g^−1^.h^−1^)	1.14 (0.41)	0.99 (0.22)	ns
Cellobiohydrolase (CEL, µmol.g^−1^.h^−1^)	0.19 (0.07)	0.15 (0.05)	ns
N-acetyl-β-D-glucosaminidase (NAG, µmol.g^−1^.h^−1^)	0.45 (0.31)	0.15 (0.05)	***
Phosphatase (PME, µmol.g^−1^.h^−1^)	1.75 (0.57)	1.27 (0.28)	**
Leucine-aminopeptidase (LEU, µmol.g^−1^.h^−1^)	0.03 (0.01)	0.01 (0.01)	***
BG/PME	0.66 (0.10)	0.80 (0.13)	**
BG/NAG	3.3 (1.6)	7.3 (2.5)	**
BG/LEU	40.9 (13.4)	85.2 (33.3)	***

Statistical significances are marked by asterisks as follows: *p < 0.05, **p < 0.01, ***p < 0.001, ns – not significant, nc – not calculated; ^a^CUE and NUE were calculated from ^a^vailable C (DOC) and N (NO_3_^−^ + NH_4_^+^).

Both soils had similar rates of basal respiration, but spruce soil had a lower microbial biomass (C_mic_, N_mic_), resulting in higher specific respiration of ~12 nmol C.g C_mic_^−1^.d^−1^ rather than ~9 nmol C.g C_mic_^−1^.d^−1^ in the beech soil (Table 2). The amount of N in microbial biomass (N_mic_) was proportionally lower compared to microbial carbon (C_mic_) in the spruce soil, increasing the C_mic_/N_mic_ ratio and pointing to the higher abundance of fungi in said soil. This was supported by qPCR data, which confirmed the higher fungal SSU gene copy numbers here (Table 3).

**Table 3:** Microbial community properties of beech (n = 16) and spruce (n = 16) soils.

	Beech	Spruce	
Chao1 index (prokaryota)	3028 (370)	2255 (469)	***
Chao1 index (fungi)	100 (53)	139 (92)	ns
Observed species (prokaryota)	1977 (127)	1553 (182)	***
Observed species (fungi)	64 (26)	77 (34)	ns
Bacterial abundance (SSU gene copies.ngDNA^−1^)	6.7 × 10^4^ (2.6 × 10^4^)	11.7 × 10^4^ (4.9 × 10^4^)	**
Archaeal abundance (SSU gene copies.ngDNA^−1^)	2.0 × 10^3^ (1.1 × 10^3^)	0.9 × 10^3^ (1.0 × 10^3^)	**
Fungal abundance (SSU gene copies.ngDNA^−1^)	0.3 × 10^4^ (0.4 × 10^4^)	0.9 × 10^4^ (0.7 × 10^4^)	*
F/B ratio	0.05 (0.06)	0.08 (0.05)	ns
Proteo/Acido ratio	1.6 (0.4)	1.3 (0.3)	**

Statistical significances are marked by asterisks as follows: *p < 0.05, **p < 0.01, ***p < 0.001, ns – not significant.

The ratios of microbial carbon (C_mic_) to the dissolved and total C (C_mic_/DOC, C_mic_/C_TOT_, Table 2) were lower in the spruce soil, which was in line with the lower C use efficiency (CUE) of the spruce microbial community (Table 2). It also corresponded with the 38% higher spruce specific respiration. Conversely, nitrogen use efficiency (NUE) was ~30% greater in the spruce soil compared with the beech (Table 2).

The spruce soil, furthermore, displayed a more substantial difference between biomass C_mic_/N_mic_ and available resource DOC/DN (20 vs. 6, for spruce and beech, respectively), which means that microbes in the spruce soil must cope with much larger stoichiometric difference when utilizing the C and N resources.

Total enzymatic activity was lower in the spruce soil (Table 2). The pronounced differences were mainly in N and P acquiring enzymes that significantly influenced C/N and C/P enzyme ratios. C/N and C/P enzyme ratios were much higher in the spruce soil (Table 2), suggesting proportionally higher C to N mining from complex resources.

Most of the measured processes of microbial activity in the beech soil correlated with chemical properties, while in the spruce surprisingly, the only important relationship found was for C_mic_ and soil pH (Table S1). Basal respiration in beech soil correlated positively with the available C and N (C_TOT_, N_TOT_, DOC and NH_4_^+^), microbial C (C_mic_), and net ammonification. Similarly, C_mic_ and N_mic_ in the beech soil were both positively affected by soil C and N availability, mainly C_TOT_, N_TOT_, DOC, net ammonification and NH_4_^+^ (Table S1). In the spruce, microbial C was related only to available NH_4_^+^. A strong correlation with soil pH and microbial C and N was found also for BG, PME and NAG activity in the beech soil (data not shown). These results suggest that the beech soil exhibited a much closer connection between soil properties, organic matter transformation and microbial activity.

### Composition of bacterial, archaeal and fungal communities

**Figure 1:**
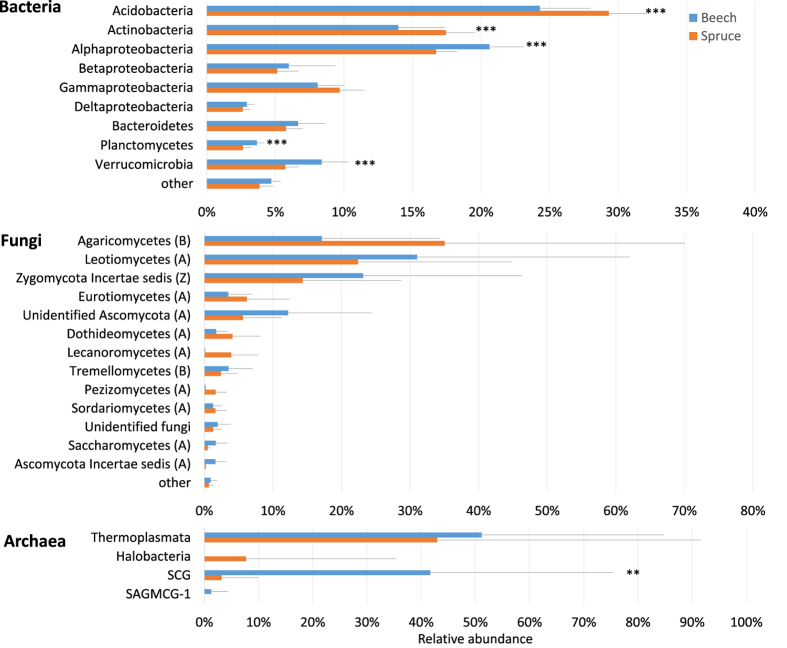
Differences in the composition of bacterial, fungal and archaeal communities between the beech (n = 16) and spruce (n = 16) soils. Only those phyla and classes with more than 1% of relative abundance are shown. Statistical significances are marked by asterisks as follows: *p < 0.05, **p < 0.01, ***p < 0.001.

Bacterial communities in both soils were dominated by three phyla: Acidobacteria, Actinobacteria and Proteobacteria which together represented 65% and 63% of assigned sequences in the beech and spruce soil, respectively (Fig. 1). Acidobacteria and Actinobacteria were more abundant in the spruce soil (29% and 17%) than in the beech (24% and 14%). On the other hand, the beech soil sustained larger community of Proteobacteria, Planctomycetes and Verrucomicrobia (21%, 4%, and 8% compared with 17%, 3% and 6% for the spruce soil). Except Halobacteria, all detected Archaeal classes: Thermoplasmata, Soil crenarcheal group (SCG) and South African gold mine crenarchaeal group 1 (SAGMCG-1) were more numerous in the beech soil. The most pronounced difference showed archaeal class SCG from Thaumarcheota phylum, which comprised 41% in the beech soil but only 4% in the spruce soils (Fig. 1). Using Bray-Curtis distances and the Ward linkage between OTUs, 4 large OTU clusters showed distinct abundance patterns between the spruce and beech prokaryotic communities (Fig. S8). The cluster I and cluster II represented the most abundant OTUs assigned mainly to Acidobacteriaceae (Subgroup 1), showing significantly higher abundances in the spruce community. In contrast, other families of Xanthobacteraceae (Alphaproteobacteria), Bradyrhizobiaceae (Alphaproteobacteria), Opitutaceae (Verrucomicrobia) and Acidobacteria (Subgroup 3) formed larger proportions in the beech community. Deeper taxonomical analyses revealed that the 10 most abundant bacterial genera represented on average 43% and 36% in the spruce and beech prokaryotic communities, respectively (Fig. S9). Uncultured Acidobacteriaceae (Subgroup 1) bacterium, Acidothermus, Acidobacterium and Rhodanobacter figured more prominently in the spruce community. On the other hand, uncultured bacterium from Verrucomicrobia phylum, uncultured bacterium from Acidobacteria subgroup 2 and uncultured Xanthobacteriaceae bacterium were more abundant in the beech prokaryotic community.

The beech and spruce soils also differed in their alpha diversity of prokaryotic and fungal communities. The former possessed greater prokaryotic richness as indicated by Chao1 index (beech soil – 1881; spruce soil – 1389). However, the opposite was true for the fungal community, though the difference was not significant (Table 3).

**Figure 2:**
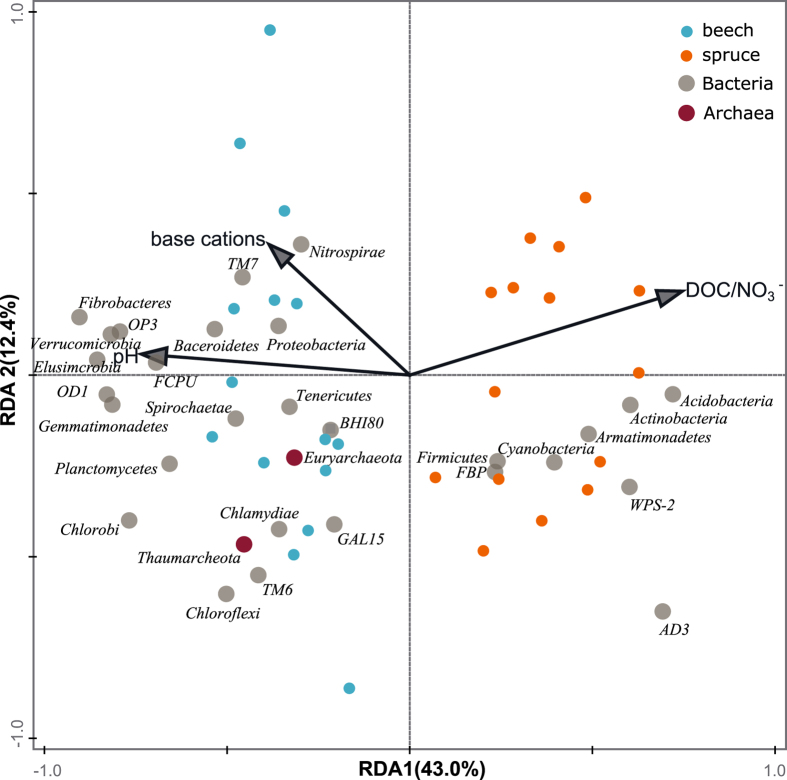
Redundancy analyses (RDA) of prokaryotic community. RDA of relative OTU abundances of prokaryotic phyla in the beech (n = 16) and spruce (n = 16) soils. The relation of the environmental variables to the prokaryotic community composition is shown. Each point represents an individual soil sample used in the analysis. The direction and length of arrows show the correlational strength between the abundance of each prokaryotic phylum and environmental variable. RDA1 axis explained 43.0% and RDA2 explained 12.4% of variability in prokaryotic community composition.

The beech and spruce prokaryotic communities were clearly separated by the first RDA axis, explaining 43% of the variability in prokaryotic communities (Fig. 2). Soil pH, DOC:NO_3_^−^ ratio and base cations (Mg^2+^  + Ca^2+^) were the three environmental explanatory variables with significant effects. The relative abundance of Acidobacteria and Actinobacteria correlated positively with the higher DOC:NO_3_^−^ ratio and with the lower pH, while the less acidic, NO_3_^−^ rich beech soil (Fig. 2, Table 1) supported greater numbers of Proteobacteria, Plantomycetes and Verrucomicrobia. These five most abundant phyla were the main phyla separating the beech from the spruce soil (Fig. 2). The separation of spruce and beech communities was additionally confirmed by metric and nonmetric multidimensional scaling (MDS, NMDS) analysis using Bray-Curtis distances. The MDS and NMDS analyses (Figs S10 and S12) using Bray-Curtis distances, which were done on OTU level, showed similar separation as RDA analyses using Euclidean distances (Figs 2 and S11).

Due to the very high variability among the samples, differences of major fungal phyla were not statistically significant (Fig. 1, Figs S11 and S12a,b). The spruce soil levels of Agaricomycetes (Basidiomycota) were higher (35%) than those for the beech soil (17%) while opposite was true for Leotiomycetes (Ascomycota) (beech soil – 32%; spruce soil – 25%). Analyses of fungal life strategies showed that saprotrophic fungi were more abundant in the beech soil (37%) than in the spruce soil (24%), while the opposite was true for ectomycorrhizal fungi, which amounted to 16% in the spruce and 9% in the beech soil, respectively (Fig. S6).

Both soils differed in prokaryotic functional potential calculated by PICRUSt^57^ algorithms. Principal component analysis of functional potential separated the beech and spruce microbial communities along the first PCA axis, showing differences in functional potential in the beech and spruce microbial communities, however with more functionally similar samples than the taxonomic differences (Figs 1 and S5). PICRUSt functional analysis showed that the beech and spruce microbial communities differed mainly in abundance of transporting systems, N metabolism and peptidases (Fig. S4). It supported measured differences in N enzymatic activity and showed more copiotrophic nature of the beech community (i.e. more transporting systems and more processes connected with membrane like respiration).

**Figure 3:**
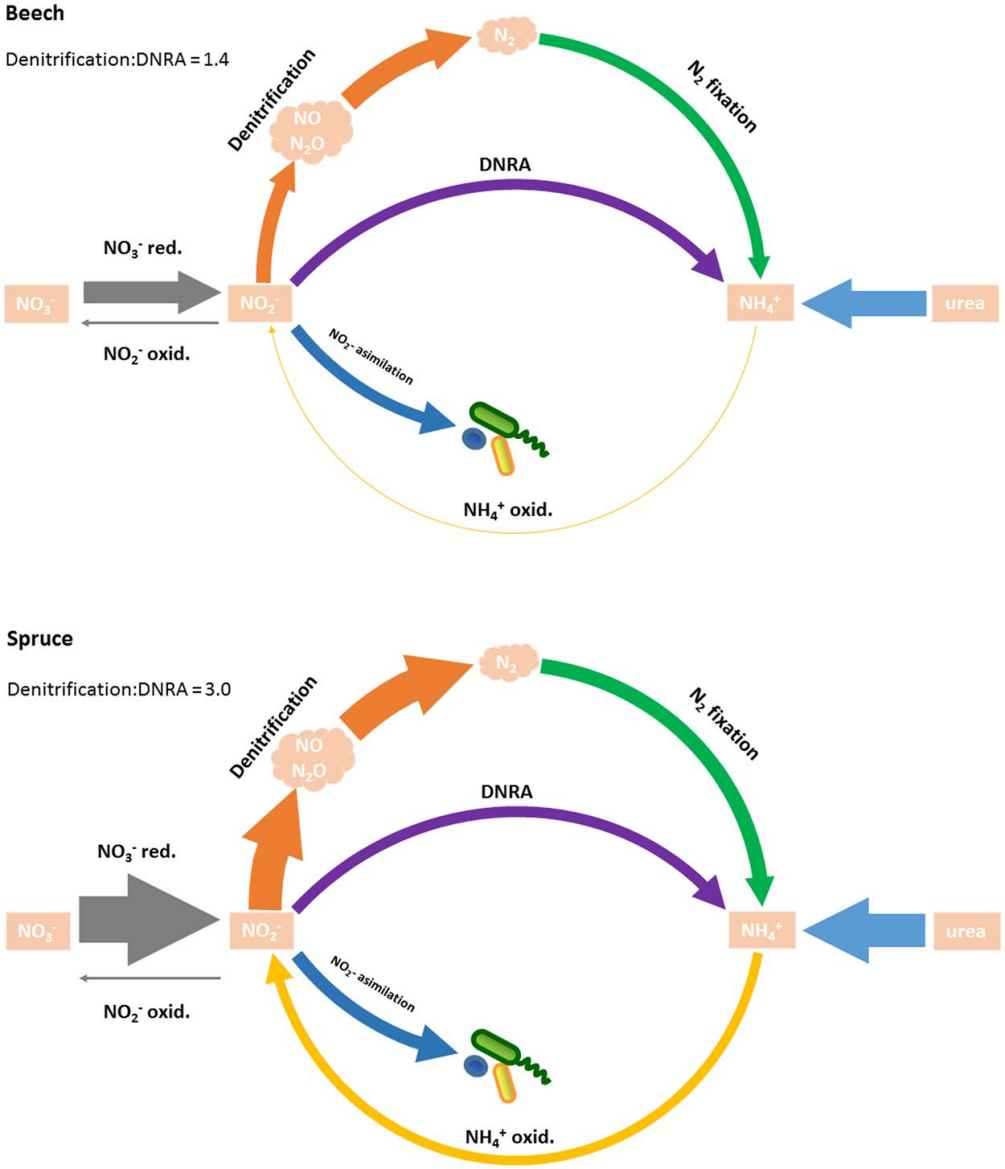
Conceptual scheme of main N cycle pathways in the spruce and beech soils. Nitrogen transformation processes are depicted with different colors. Pathways are based on functional assignment (RDP FunGene database, Table S2). Thickness of the arrows corresponds to absolute abundances of assigned bacterial functional guilds recalculated by qPCR.

For the OTUs, which were classified at the genus level, it was also possible to assign functional genes based on RDP FunGene database (Table S2). By combining SSU qPCR data and relative abundances of assigned bacterial genera, we were able to quantitatively identify the main N transformation pathways (Fig. 3). It allows us to reconstruct and identify differences in the N cycle in the beech and spruce microbial communities (Figs 3 and S7). Microorganisms capable of nitrate reduction (napA, narG genes), denitrification (nirK, nirS, norB, nosZ genes), N_2_ fixation (nifH gene) and NH_4_^+^ oxidation (amoA) were more abundant in the spruce than in the beech community. The abundance of microorganisms capable of dissimilative nitrate reduction to ammonium (DNRA, nrfA gene), however, did not differ between the beech and spruce community. Therefore, in the spruce soil, the higher proportion of NO_3_^−^ can be potentially lost via denitrification (denitrification to DNRA ratio is 3) while in the beech soil, the higher proportion of NO_3_^−^ can be recycled in the soil system and the microbial community most likely via DNRA (denitrification to DNRA ratio is 1.4).

**Figure 4:**
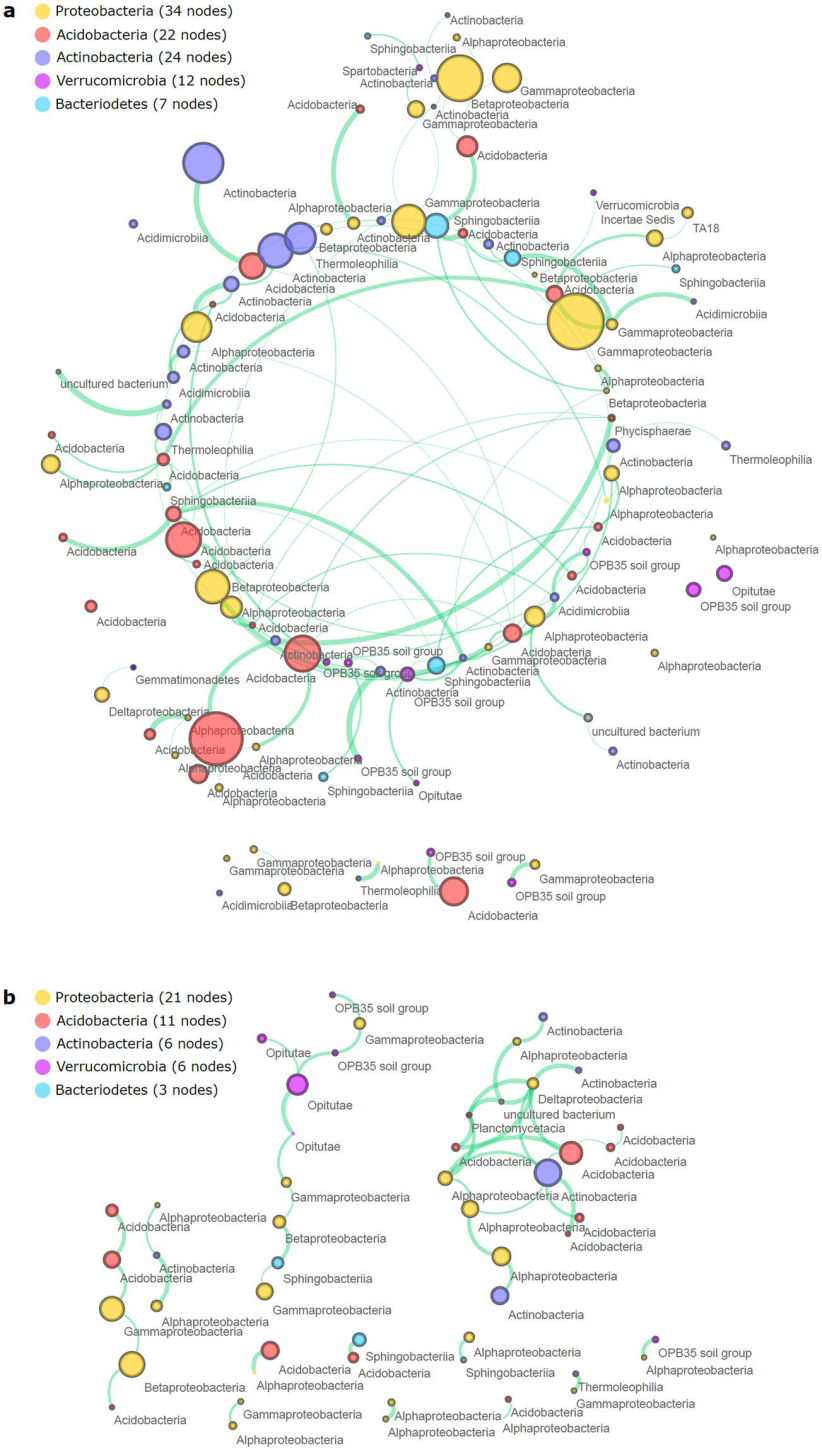
OTU network analyses of the beech (**a**) and spruce (**b**) prokaryotic communities. Each OTU (node) is colored by the phylum it belongs to. Labels of nodes shows respective bacterial or archaeal classes. The size of node corresponds to the average abundance of each OTU. Green color of edges shows positive relationship (i.e. co-presence of OTUs) and red edge color shows negative relationship (i.e. mutual exclusion of OTUs).

In the next step, network analysis was performed to describe relationships between distinct OTUs in the beech and spruce prokaryotic communities and identify the most important OTUs (i.e. keystone OTUs). We found dramatic differences between the beech and spruce prokaryotic networks (Fig. 4a,b, Table 4, Fig. S14). While in the beech prokaryotic network the number of relationships was low and infrequent (more separate groups of interacting OTUs with only 51 significantly interacting OTUs), the opposite was true for the spruce network (103 significantly interacting OTUs with dense interacting network). The network analyses with the support of 100 instances of bootstrapping revealed 145 significant interactions (edges) in the spruce prokaryotic community while in in the beech community only 45 showed very intense interconnection of prokaryotes (Fig. 4b). Interestingly, all interactions in the beech and spruce prokaryotic communities were positive (co-presence). We specifically identified 7 potential keystone OTUs in both communities (Table S2). The spruce potential keystones were mainly from the phylum Acidobacteria (4 out of 7 keystones) while the beech keystones recruited from Proteobacteria (3 out of 7 keystones). In the beech soil, microorganisms capable of DNRA (Acidothermus, Planctomyces, Opitutus) were among the potential keystone OTUs, but in the spruce community nitrate reducing (Acidobacterium) and denitrifying microorganisms (Acidothermus, Herminiimonas) dominated (Table 4).

**Table 4:** Keystone OTUs in beech and spruce prokaryotic community.

Phylum	Genus	Degree	Betweenness Centrality	Closeness Centrality	Function in N cycle
**Potential keystone species in beech prokaryotic community**					
Proteobacteria	*uncultured bacterium*	6	0,413	0,516	—
Actinobacteria	*Acidothermus*	6	0,385	0,516	denitrifier
Planctomycetes	*Planctomyces*	4	0,101	0,432	DNRA, nitrate reducer
Acidobacteria	*uncultured bacterium*	4	0,239	0,471	—
Proteobacteria	*uncultured bacterium*	4	0,138	0,471	—
Proteobacteria	*uncultured*	3	0,233	0,400	—
Verrucomicrobia	*Opitutus*	3	0,639	0,409	DNRA, nitrate reducer
**Potential keystone species spruce prokaryotic community**					
Acidobacteria	*Acidobacterium*	10	0,163	0,290	nitrate reducer
Actinobacteria	*uncultured Actinomycetales*	9	0,270	0,321	—
Acidobacteria	*Candidatus Solibacter*	7	0,133	0,271	—
Acidobacteria	*Candidatus Solibacter*	7	0,224	0,311	—
Actinobacteria	*Acidothermus*	7	0,118	0,294	denitrifier
Proteobacteria	*Herminiimonas*	6	0,099	0,295	denitrifier
Acidobacteria	*Candidatus Koribacter*	6	0,095	0,281	—

Basic properties of keystone species (degree, betweenness centrality, closeness centrality) are shown. Taxonomic classification is based on Silva 119 database. Annotated function of known bacterial genera in N cycle are shown in the last column.

The microbial networks of the beech and spruce fungal community showed similar trends as prokaryotic ones (Fig. S13a,b). The spruce fungal network had more interacting species (n. of nodes = 117) and also more connections (n. of edges = 498) than beech community which had 51 nodes and 246 edges, respectively. Species of Mortierella (Zygomycota) and Cryptococcus (Basidiomycota) were the most interacting in beech and spruce community, respectively.

## Discussion

Our study makes use of the comparison of two closely located forests (1 km of each other) which are situated on the same acid bedrock, have similar climate and deposition history but differ in tree dominants. In the spruce forest the nitrates in the soil leachate have almost diminished since the 1990s^7^, while in the beech forest the nitrate leaching still continues. Soil solution chemistry revealed less acidic conditions in the beech compared to the spruce forest, which was surely the effect of litter type and long-term lower dry deposition^61^. Soil prokaryotic communities, not fungi, differed in their composition substantially between the spruce and beech, and their structures were to a certain extent linked to different soil C and N biogeochemistry.

The main difference between the spruce and beech microbial community was found in the structure of prokaryotes (Fig. 2). The spruce prokaryotic community had lower species diversity but more abundant than the beech community (Table 3), which corresponds to the published data of similar biomes^62^. It comprised three main bacterial phyla: Acidobacteria, Proteobacteria and Actinobacteria (Fig. 1). Although Archaea formed only a minor population compared to bacteria, especially in the spruce forest (Table 3), a considerable difference was found for the Soil Crenarchaeotic Group (SCG) from the phylum Thaumarchaeota that comprises over 40% of relative abundance in the beech soil compared to only a negligible part of the spruce archaeal community (Fig. 1).

The beech and spruce fungal communities were not separated (Fig. S11) and did not differ significantly even in relative abundances (Fig. 1). One reason could be that our sampling strategy, in which we wanted to pinpoint the most pronounced functional differences in the microbial community^63^ and thus sampled the most active forest floor layer as a mixture (Of + Oh + A), diminished the differences between the beech and spruce fungal communities, usually showing the depth stratification. Despite this, we found prevailing saprotrophic strategy over the mycorrhizal in both fungal communities (Fig. S6), which is most probably the effect of former N deposition^64^. The fungal community composition comprised of Ascomycota, Basidiomycota and Zygomycota (Fig. 1), which is in line with globally described patterns of temperate forests^65^.

Despite only a narrow pH range and little difference between both forests (~0.2 pH units), pH and base cation concentration still explained most of the differences between the spruce and beech prokaryotic community (Fig. 2). Since soil base saturation is related to soil pH through the exchange of H^+^ between organo-mineral complexes and soil solution, it is reasonable to assume that both parameters shaped the composition of the prokaryotic community simultaneously. Our results conform with the general actuality that soil pH is one of the strongest predictors of microbial community composition^12,66–70^ and that Acidobacteria often predominate in acidic soils^71^.

In our study, lower pH was always connected with a higher proportion of Acidobacteria and Actinobacteria (Fig. 2), representing together ~47% and ~37% of the spruce and beech bacteria, respectively (Fig. 1). This was within a range of other forest studies^62,72^. In both forests, the subgroup 1 was the most abundant Acidobacteria group represented mostly by genera Acidobacterium (4% and 3% for spruce and beech, respectively), Granulicella (3% and 2% for spruce and beech, respectively) and Candidatus Koribacter (1% in both soils). Acidobacterium and Granulicella were in the top 10 most abundant genera. Jones *et al*.^73^ showed that the abundance of Subgroup 1 strongly increased with decreasing soil pH. The high abundance of Subgroup 1 is most probably the reason why soil pH was the main important predictor of prokaryotic community composition.

In the beech community, the members of the phylum Proteobacteria were more abundant than in the spruce prokaryotic community. Within the Proteobacteria, the uncultured bacterium from the family Xanthobacteriaceae (Alphaproteobacteria) dominated. Bacteria from this family are moderately acidophilic. They are usually isolated from decaying organic material^74^ as well as from nutrient rich rhizosphere^75^. The next higher abundance of Verrucomicrobia and Planctomycetes in the beech community could be related, in addition to the less pronounced acidity effect^67^, to high soil N availability (Table 1). Buckley *et al*.^76^ (2006) showed that species richness of Planctomycetes correlated highly with NO_3_^−^ spatial distribution in soil, and Navarrete *et al*.^72^ concurrently found that a community of Verrucomicrobia exhibited higher abundances in soils of higher fertility.

It has been suggested that soil bacteria can be classified into copiotrophic and oligotrophic categories (corresponding to the r- and K-selected strategies)^27,68^. Although this concept can be viewed as a simplification, its application to compositional genomic data allows us to describe the ecological requirements of the soil microbial community. We expected that both forests could generally create habitats more suitable for fast-growing copiotrophs as both were exposed to high N deposition. Our data showed that the beech soil was relatively richer in available N yet at the same time much poorer in C compared to the spruce soil (Table 1). Moreover, the second most significant factor that separated the spruce and beech prokaryotic communities was the DOC/NO_3_^−^ ratio (Fig. 2). This inverse relation of both elements has been recognized as a primary predictor of soil N retention ability^10,11^. At the same time, we found a higher proportion of Proteobacteria to Acidobacteria in the beech than in the spruce microbial community (1.6 vs. 1.3, Table 3).

According to Fierer *et al*.^68^, the phylum Proteobacteria generally comprises copiotrophic taxa. They preferentially consume labile and readily available organic C, have high nutritional requirements and exhibit high growth rates when resources are abundant^68,77^. On the other hand, Acidobacteria can be considered as oligotrophs, which are slow-growing and exploit environments with lower nutrients, lower C availability or substrates of lower quality^68^. They are, therefore, able to outcompete copiotrophs in these conditions due to their higher substrate affinities and more efficient enzymatic apparatus for utilizing complex biopolymers^78^. Recent studies argue that, despite being oligotrophic, some subgroups of Acidobacteria show high relative abundances also in C rich conditions^73,80^, indicating that additional factors other than C availability drive the metabolism of various Acidobacterial subgroups. Several studies also showed that Acidobacteria decrease under elevated N conditions while Proteobacteria increase^20,27,79,80^. Our results suggest that the ratio of abundances of these large bacterial phyla may be connected with (i) soil N availability and (ii) the quality of C substrate rather than only C concentration^81,82^.

Organic matter decomposition is driven by the availability of labile C and N^83^. The DOC and DN in the soil leachate determined in our study represent a measure of labile resources, which control organic matter decomposition, including the activities of soil extracellular enzymes. Because these enzymes release low molecular weight compounds from complex biomolecules (e.g. cellulose, proteins), they also directly influence the proportions of dissolved C and N^25^. In the spruce soil, C acquiring enzymes (Table 2) were proportionally higher compared to N acquiring enzymes, which is in accordance with the measured higher DOC:DN ratio (Table 1) and suggests the decomposition of rather complex organic matter in the spruce soils. This corresponds with higher fungal abundance and also higher abundance of bacteria from the families Acidobacteriaceae subgroup 1 and Acidothermaceae, which are able to decompose complex substrates such as polyphenols and chitin and whose distribution in soils is connected with the quality of soil organic C^84^.

The ability of soil to immobilize nitrogen is closely connected with its C-to-N stoichiometry^9,10,85,86^. Although both soils showed similar net nitrification rates, nitrate concentrations were high only in the beech soil solution. It has been shown that during decomposition all organic N can be used for building microbial biomass when C is in excess^39^. Microbes can then immobilize additional mineral N from the soil solution to meet their demands^9,87^, or they can start to use nitrates in reduction processes of microbial energy metabolism (denitrification and/or dissimilative nitrate reduction to ammonia (DNRA)^4^. It might be that in C rich spruce soil with high C acquiring enzyme activity and net N mineralization, immobilization of mineral N could be more significant than in the beech soil. Concurrently, we found low C while high N use efficiencies and high specific respiration in the spruce soil supporting our assumption of possible C oversupply of spruce microbes (Table 2).

We examined the hypothesis of high nitrate immobilization in the spruce soil from the perspective of microbial community composition. Linking taxonomic classification with functional potential allowed us to identify bacterial functional guilds (e.g. N_2_ fixators, nitrifiers, denitrifiers and bacteria capable of DNRA) responsible for key transformations of mineral N. In the spruce community, the occurrence of NO_3_^−^ to NO_2_^−^ reducers was twice as high as in the beech community. However, NO_2_^−^ is a very labile compound and is immediately reduced to N gases through denitrification or via DNRA to NH_4_^+^. Our data showed that denitrifiers in the spruce community were proportionally more abundant than bacteria capable of DNRA (3:1 ratio, Fig. 3) compared to the beech l community. Marked differences were mainly in the abundance of nirK denitrifiers, whose abundance correlates closer with the available C compared to nirS denitrifiers^88^. In contrast, the beech microbial community had a lower denitrification-to-DNRA ratio (1.4:1, Fig. 3), allowing more N to be recycled back to NH_4_^+^ and possibly to NO_3_^−^ via nitrification.

The functional analyses revealed that low NO_3_^−^ concentration in the spruce soil solution may be the result of N loses through denitrification. This supports the generally observed fact that DOC/NO_3_^−^ stoichiometry regulates ecosystem NO_3_^−^ retention^11,89^. Under the high DOC/NO_3_^−^ ratio (higher than 150 ^89^), which supplies sufficient organic C to microorganisms, denitrification predominates over DNRA and N can be lost from the soil to the atmosphere. Under low DOC/NO_3_^−^,^11^, DNRA is higher or equal to denitrification and N can be recycled in the soil via NH_4_^+^.

Microbial network analyses supported the importance of denitrifiers and bacteria capable of DNRA in the soil community. Both groups were among the 10 potential keystone species of both communities (Fig. 4, Table S4). Microbes capable of DNRA (*Planctomyces*, *Opitutus*) were identified as keystone in the beech soil, while denitrifiers (*Acidothermus*, *Herminiimonas*) were instead significant players in the spruce soil. Furthermore, microbes in the spruce community showed a much higher degree of associations than the beech microbes. It points to the much higher microbial interconnection in the C rich environment, probably due to the higher substrate complexity, whose degradation needs to be synchronized by a variety of microbial functional guilds. Higher interconnection may also be an indirect result of the higher abundance of fungi, which interconnects by hyphal growth spatially distant microbes^90^. In contrast, microbes in the beech community were associated very rarely, partly because of, perhaps, their rather copiotrophic nature (quickly growing, quickly dying), and do not have, therefore, the reason and time to create more dense and interconnected microbial network.

Here we presented that the DNA sequencing approach in combination with in-silico functional analysis of microbial community structure can be a useful tool in predicting soil N biogeochemistry. Both forest soil microbial communities differed in their taxonomic diversity and functional diversity of the N cycle resulting in different potentials of soil N immobilization. We demonstrated that nitrates may be lost through denitrification in N saturated soils under recovery when organic matter decomposition is restored and the carbon availability increases^7^. Our study represents a basis for future testing of the nitrate reduction as potentially more important N immobilization process than was previously thought in acid forest soils.

## Electronic supplementary material

Supplementary Table S2

Supplementary Information
