# Author Correction: Microbial communities with distinct denitrification potential in spruce and beech soils differing in nitrate leaching

**DOI:** 10.1038/s41598-018-22998-z

**Published:** 2018-03-14

**Authors:** Jiří Bárta, Karolina Tahovská, Hana Šantrůčková, Filip Oulehle

**Affiliations:** 1 Department of Ecosystem Biology, Faculty of Science, University of South Bohemia, Branišovská 31, 370 05 České Budějovice, Czech Republic; 2 Czech Geological Survey, Department of Environmental Geochemistry and Biogeochemistry, Prague, 118 21 Czech Republic

Correction to: *Scientific Reports* 10.1038/s41598-017-08554-1, published online 29 August 2017

This Article contains an error in the legend of Figure 4.

“OTU network analyses of the beech (a) and spruce (b) prokaryotic communities. Each OTU (node) is colored by the phylum it belongs to. Labels of nodes shows respective bacterial or archaeal classes. The size of node corresponds to the average abundance of each OTU. Green color of edges shows positive relationship (i.e. co-presence of OTUs) and red edge color shows negative relationship (i.e. mutual exclusion of OTUs).”

should read:

“OTU network analyses of the spruce (a) and beech (b) prokaryotic communities. Each OTU (node) is colored by the phylum it belongs to. Labels of nodes shows respective bacterial or archaeal classes. The size of node corresponds to the average abundance of each OTU. Green color of edges shows positive relationship (i.e. co-presence of OTUs) and red edge color shows negative relationship (i.e. mutual exclusion of OTUs).”

