# Supplementary material for: Microbial communities with distinct denitrification potential in spruce and beech soils differing in nitrate leaching
Source: Sci Rep. 2017 Aug 29;7:9738. doi: 10.1038/s41598-017-08554-1 (PMC5575336; doi:10.1038/s41598-017-08554-1)
Supplement: Supplementary file 2 — Supplementary Information [file 41598_2017_8554_MOESM2_ESM.docx]

Supplementary Material for:

“Microbial communities with distinct denitrification potential in spruce and beech soils differing in nitrate leaching”

Jiří Bárta*, Karolina Tahovská, Hana Šantrůčková, Filip Oulehle

* Correspondence: Jiří Bárta: jiri.barta@prf.jcu.cz

Table S1: Pearson correlation coefficients (r) of selected soil and microbial parameters in the beech (B, n = 16) and spruce (S, n = 16) soils.

|  | pH | | |  | C_TOT_ | | |  | N_TOT_ | | |  | C_mic_ | | |  | N_mic_ | | |  | DOC | | |  | respiration | | |  | N-NH_4_^+^ | | |  | N-NO_3_^-^ | | |  |  |
| --- | --- | --- | --- | --- | --- | --- | --- | --- | --- | --- | --- | --- | --- | --- | --- | --- | --- | --- | --- | --- | --- | --- | --- | --- | --- | --- | --- | --- | --- | --- | --- | --- | --- | --- | --- | --- | --- |
|  | **B** |  | **S** |  | **B** |  | **S** |  | **B** |  | **S** |  | **B** |  | **S** |  | **B** |  | **S** |  | **B** |  | **S** |  | **B** |  | **S** |  | **B** |  | **S** |  | **B** |  | **S** |  |  |
| pH | **-** |  | **-** |  | ns |  | ns |  | ns |  | ns |  | ns |  | **0,7** | ****** | ns |  | ns |  | **0,8** | ****** | ns |  | **0,5** |  | ns |  | **0,8** | ******* | ns |  | **-0,7** | ****** | ns |  |  |
| C_TOT_ |  |  |  |  | **-** |  | **-** |  | **0,9** | ******* | ns |  | **0,7** | ****** | ns |  | **0,6** | ***** | ns |  | **0,7** | ****** | ns |  | **0,8** | ******* | ns |  | **0,8** | ******* | ns |  | ns |  | ns |  |  |
| N_TOT_ |  |  |  |  |  |  |  |  | **-** |  | **-** |  | **0,7** | ****** | ns |  | ns |  | ns |  | ns |  | ns |  | **0,8** | ******* | ns |  | **0,7** | ****** | ns |  | ns |  | ns |  |  |
| C_mic_ |  |  |  |  |  |  |  |  |  |  |  |  | **-** |  | **-** |  | **0,7** | ****** | **0,9** | ******* | **0,6** | ***** | ns |  | **0,8** | ******* | ns |  | **0,8** | ******* | **0,7** | ****** | ns |  | ns |  |  |
| N_mic_ |  |  |  |  |  |  |  |  |  |  |  |  |  |  |  |  | **-** |  | **-** |  | **0,6** | ***** | ns |  | ns |  | ns |  | **0,5** | ***** | **0,6** | ***** | ns |  | ns |  |  |
| DOC |  |  |  |  |  |  |  |  |  |  |  |  |  |  |  |  |  |  |  |  | **-** |  | **-** |  | **0,6** | ***** | ns |  | **0,8** | ******* | **0,8** | ****** | ns |  | ns |  |  |
| respiration |  |  |  |  |  |  |  |  |  |  |  |  |  |  |  |  |  |  |  |  |  |  |  |  | **-** |  | **-** |  | **0,8** | ******* | ns |  | ns |  | ns |  |  |
| N-NH_4_^+^ |  |  |  |  |  |  |  |  |  |  |  |  |  |  |  |  |  |  |  |  |  |  |  |  |  |  |  |  | **-** |  | **-** |  | ns |  | **0,6** | ****** |  |
| N-NO_3_^-^ |  |  |  |  |  |  |  |  |  |  |  |  |  |  |  |  |  |  |  |  |  |  |  |  |  |  |  |  |  |  |  |  | **-** |  | **-** |  |  |
|  |  |  |  |  |  |  |  |  |  |  |  |  |  |  |  |  |  |  |  |  |  |  |  |  |  |  |  |  |  |  |  |  |  |  |  |  |  |

Correlation coefficients are given and their significances are marked by asterisks as follows: *p<0.05, **p<0.01, ***p<0.001, ns – not significant.

Table S3: Coverage of primer pair (515FB-GTGYCAGCMGCCGCGGTAA and 806RB-GGACTACNVGGGTWTCTAAT) which was tested in-silico by TestPrime algorithm on ARB-Silva database (release 128). The primer pair 515FB/806RB covers almost uniformly all major bacterial and archaeal phyla.

| **Domain,phylum** | **coverage** |
| --- | --- |
| **Bacteria** | **87.5%** |
| AC1 | 69.1% |
| Acetothermia | 93.5% |
| Acidobacteria | 91.8% |
| Actinobacteria | 81.1% |
| Aerophobetes | 3.2% |
| Aminicenantes | 88.6% |
| Aquificae | 86.9% |
| Armatimonadetes | 86.4% |
| Atribacteria | 93.6% |
| Bacteroidetes | 88.6% |
| BJ-169 | 91.7% |
| BP4 | 100.0% |
| BRC1 | 85.6% |
| Caldiserica | 5.3% |
| Calescamantes | 0% |
| Candidatus Berkelbacteria | 46.6% |
| Chlamydiae | 18.8% |
| Chlorobi | 55.8% |
| Chloroflexi | 53.5% |
| Chrysiogenetes | 100.0% |
| Cloacimonetes | 83.2% |
| CPR2 | 72.2% |
| Cyanobacteria | 80.9% |
| Deferribacteres | 87.8% |
| Deinococcus-Thermus | 92.2% |
| Dictyoglomi | 88.9% |
| Elusimicrobia | 88.8% |
| FBP | 15.0% |
| FCPU426 | 75.8% |
| Fervidibacteria | 100.0% |
| Fibrobacteres | 85.5% |
| Firmicutes | 88.6% |
| FL0428B-PF49 | 73.3% |
| Fusobacteria | 88.3% |
| GAL15 | 88.6% |
| Gemmatimonadetes | 88.1% |
| GN01 | 72.4% |
| Gracilibacteria | 77.1% |
| Hydrogenedentes | 88.8% |
| Ignavibacteriae | 89.6% |
| KSB3 Modulibacteria | 79.4% |
| Latescibacteria | 91.1% |
| LCP-89 | 100.0% |
| Lentisphaerae | 82.5% |
| Marinimicrobia SAR406 clade | 72.8% |
| MD2896-B216 | 33.3% |
| Microgenomates | 1.3% |
| Nitrospinae | 92.8% |
| Nitrospirae | 89.8% |
| Omnitrophica | 62.1% |
| Parcubacteria | 4.5% |
| PAUC34f | 60.1% |
| Peregrinibacteria | 86.1% |
| Planctomycetes | 82.8% |
| Poribacteria | 11.8% |
| Proteobacteria | 91.8% |
| RBG-1 Zixibacteria | 87.2% |
| RsaHF231 | 85.7% |
| Saccharibacteria | 3.9% |
| SBR1093 | 88.3% |
| Spirochaetae | 71.9% |
| SR1 Absconditabacteria | 84.2% |
| Synergistetes | 89.2% |
| TA06 | 75.0% |
| Tectomicrobia | 89.0% |
| Tenericutes | 84.2% |
| Thermodesulfobacteria | 93.2% |
| Thermotogae | 93.2% |
| TM6 Dependentiae | 92.9% |
| Verrucomicrobia | 87.5% |
| WA-aaa01f12 | 75.0% |
| WS1 | 82.2% |
| WS2 | 84.6% |
| WS6 | 0% |
| WWE3 | 0% |
| **Archaea** | **85.4%** |
| Aenigmarchaeota | 33.9% |
| Aigarchaeota | 87.6% |
| AK8 | 86.7% |
| Altiarchaeales | 0% |
| Ancient Archaeal Group (AAG) | 0% |
| Bathyarchaeota | 81.1% |
| Candidate division YNPFFA | 61.7% |
| Crenarchaeota | 89.1% |
| Diapherotrites | 5.0% |
| Euryarchaeota | 87.9% |
| Hadesarchaea | 84.7% |
| Korarchaeota | 46.3% |
| Lokiarchaeota | 85.3% |
| Marine Benthic Group E | 88.4% |
| Marine Hydrothermal Vent Group 1 (MHVG-1) | 91.7% |
| Marine Hydrothermal Vent Group 2 (MHVG-2) | 0% |
| Marine Hydrothermal Vent Group (MHVG) | 30.5% |
| Miscellaneous Euryarchaeotic Group (MEG) | 6.8% |
| MSBL1 | 84.8% |
| Nanoarchaeota | 0% |
| Nanohaloarchaeota | 0% |
| Parvarchaeota | 68.8% |
| pCIRA-13 | 0% |
| pMC2A209 | 20.0% |
| Thaumarchaeota | 91.3% |
| TVG8AR30 | 0% |
| Woesearchaeota (DHVEG-6) | 80.5% |
| WSA2 | 77.0% |

**Figure S1**

Nacetin sampling site with the depicted spruce (50°35´26´´N, 13°15´14´´E) and beech (50°35´22´´N, 13°16´07´´E) stands. The site is located on the borders with Germany in the so called “Black Triangle” with the highest acid depositions in Central Europe. Detailed maps of sites were provided by Dr. Filip Oulehle and published in Oulehle *et al.* (2007). Map of the Czech Republic was created in InkScape 0.91 software https://inkscape.org/en/ by Dr. Jiří Bárta.

**Figure S2**

Representative picture from the sampling sites. Within each site 16 randomly distributed plots (3x3m) were established. On the beech site, LI-COR fully automated chambers for in-situ CO_2_ measurement can be seen.

**Figure S3**

Wet deposition of sulphur (top) and nitrogen (bottom) in the years 1985 (left), 1995 (center) and 2008 (right) based on the EMEP model (http://www.emep.int, accessed 1 March 2011). Maps were generated by online application on EMEP site. Data were adopted from Oulehle *et al.* (2011). Re-use of content on the EEA website for commercial or non-commercial purposes is permitted free of charge, provided that the source is acknowledged http://www.eea.europa.eu/legal/copyright). Copyright holder: United Nations Economic Commission for Europe (UNECE). http://www.eea.europa.eu/data-and-maps/data/modelled-deposition-estimates-for-sulphur-and-nitrogen-from-the-emep-unified-model-on-the-standard-emep-grid-resolution-of-approx-50km-x-50km

**Figure S4**

Extended error bar showing PICRUSt analyses (for details see Methods section) with significant functional differences between the beech and spruce prokaryotic communities. Significantly more abundant function in the beech community is above the dashed line while significantly more abundant function in the spruce community is below the dashed line. Bar charts on the left side represent abundances of functional annotations based on Kyoto Encyclopedia of Genes and Genomes (KEGG) database.

**Figure S5**

Principal component analyses (PCA) of functional diversity determined by PICRUSt algorithm. KEGG functional annotation with relative abundances on functional level 3 were used for calculating distances between samples.

**Figure S6**

Fungal life strategies compared between the beech and spruce soil. OTUs which were assigned at the genus level of taxonomy were assigned to life strategies according the Tedersoo *et al.* (2014).

**Figure S7**

Radial graph showing distribution of main microbial functional genes in the beech and spruce microbial communities as annotated by Ribosomal Database Project, Fungene database (Fish *et al.*, 2013).

**Figure S8**

Prokaryotic OTU heatmap was created based on the rarified OTU table to 4,500 sequences per sample. Bray-Curtis distances were calculated for OTUs and samples using the Ward clustering algorithm. Four distinct OTU clusters were identified with distinct abundance patterns between the spruce and beech prokaryotic communities.

**Figure S9**

Top 10 most abundant genera of the spruce and beech prokaryotic communities.

**Figure S10**

(**a**) Multidimensional scaling (MDS) and (**b**) nonmetric multidimensional scaling (NMDS) analysis of prokaryotic community (OTU level) using Bray-Curtis distances between microbial communities in different samples.

**Figure S11**

Redundancy analyses (RDA) of fungal community. RDA analyses of relative OTU abundances of fungal phyla in the beech (n = 16) and spruce (n = 16) soils. The relation of the environmental variables to the fungal community composition is shown. Each point represents an individual soil sample used in the analysis. The direction and length of arrows show the correlational strength between the abundance of each prokaryotic phylum and environmental variable. RDA1 axes explained 30.4% and RDA2 explained 16.5% of variability in fungal community composition. None of the selected environmental variables explained significantly the variability in fungal community composition.

**Figure S12**

(**a**) Multidimensional scaling (MDS) and (**b**) nonmetric multidimensional scaling (NMDS) analysis of fungal community (OTU level) using Bray-Curtis distances between microbial communities in different samples.

**Figure S13**

OTU network analyses of the beech (a) and spruce (b) fungal communities. Each OTU (node) is colored by the fungal phyla it belongs to. Labels of nodes shows respective fungal genera. The size of node corresponds to the average abundance of each OTU. Green color of edges shows positive relationship (i.e. co-presence of OTUs) and red edge color shows negative relationship (i.e. mutual exclusion of OTUs). In contrast to prokaryotic network, several parameters of CoNet settings were modified (-minoccur = 4, min p value = 0.01).

**Figure S14**

OTU network analyses of the beech (a) and spruce (b) prokaryotic communities. Each OTU (node) is colored by the prokaryotic phyla it belongs to. Labels of nodes shows respective prokaryotic orders. The size of node corresponds to the average abundance of each OTU. Green color of edges shows positive relationship (i.e. co-presence of OTUs) and red edge color shows negative relationship (i.e. mutual exclusion of OTUs. The significance of the edge scores was tested on alpha level of 0.01.

**Bárta *et al*., Figure S1**


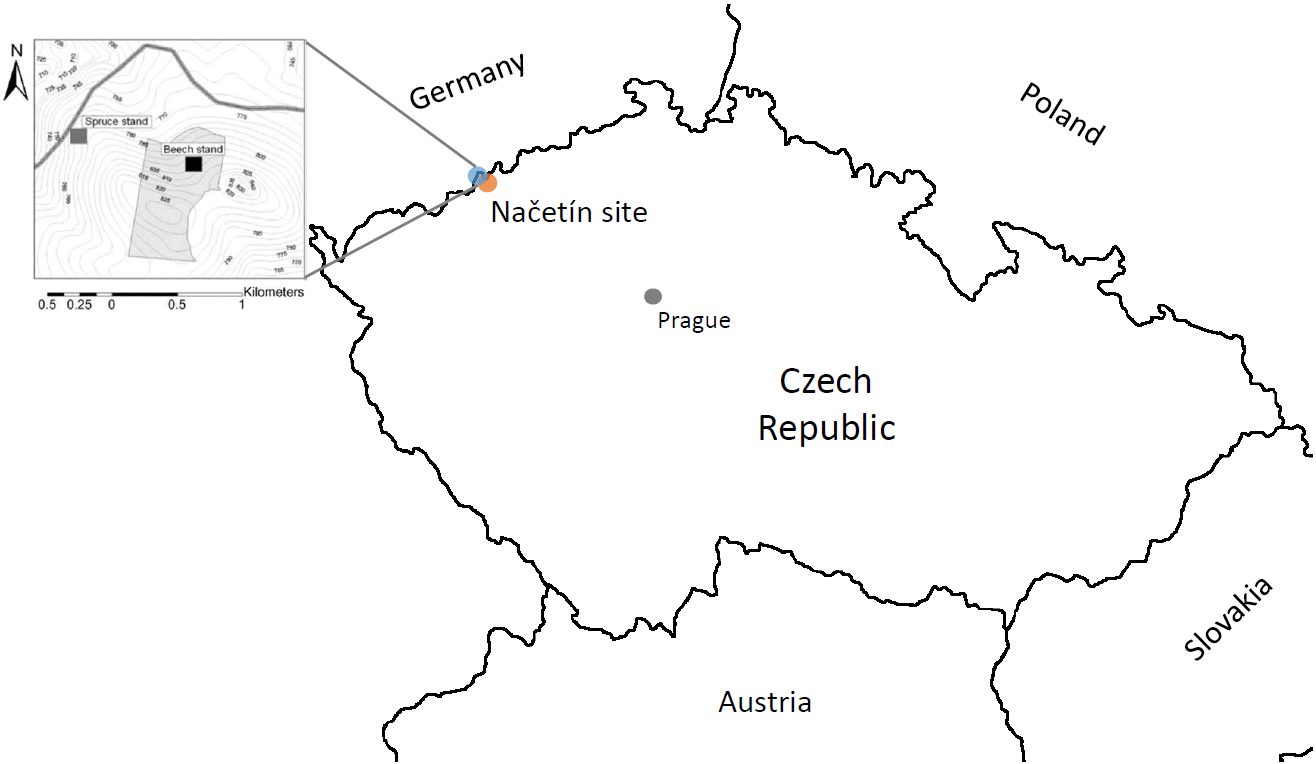


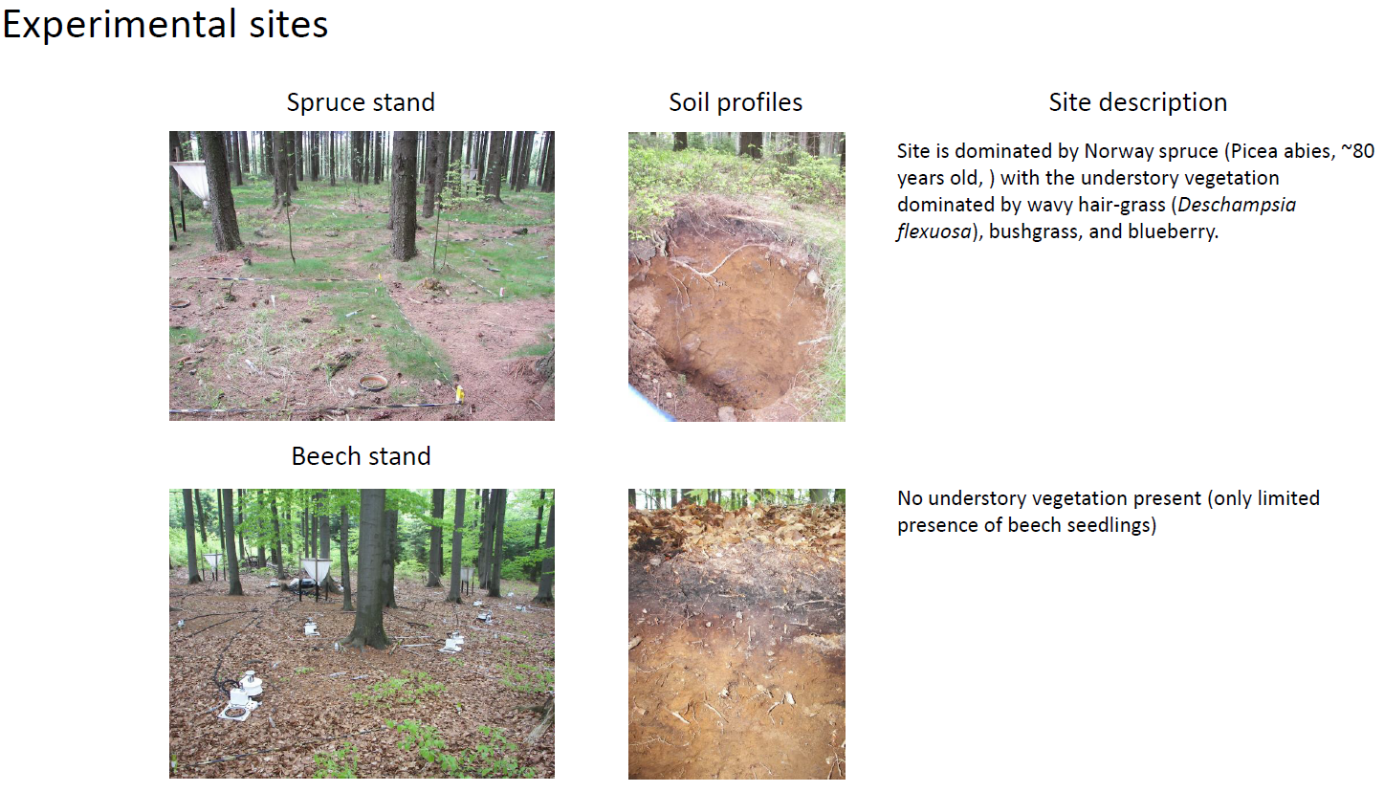
**Bárta *et al*., Figure S2**

**Bárta *et al*., Figure S3**


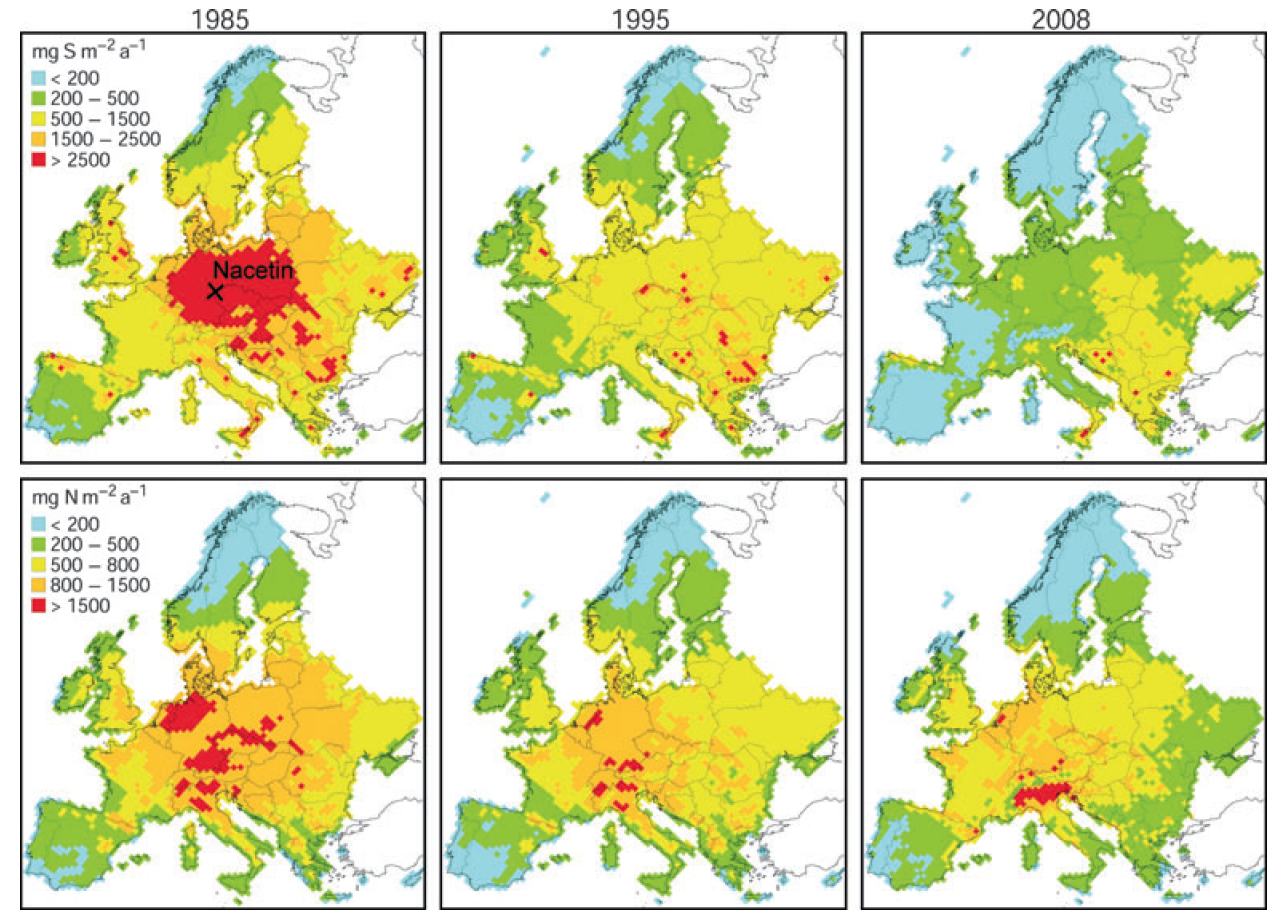


**Bárta *et. al*, Figure S4**


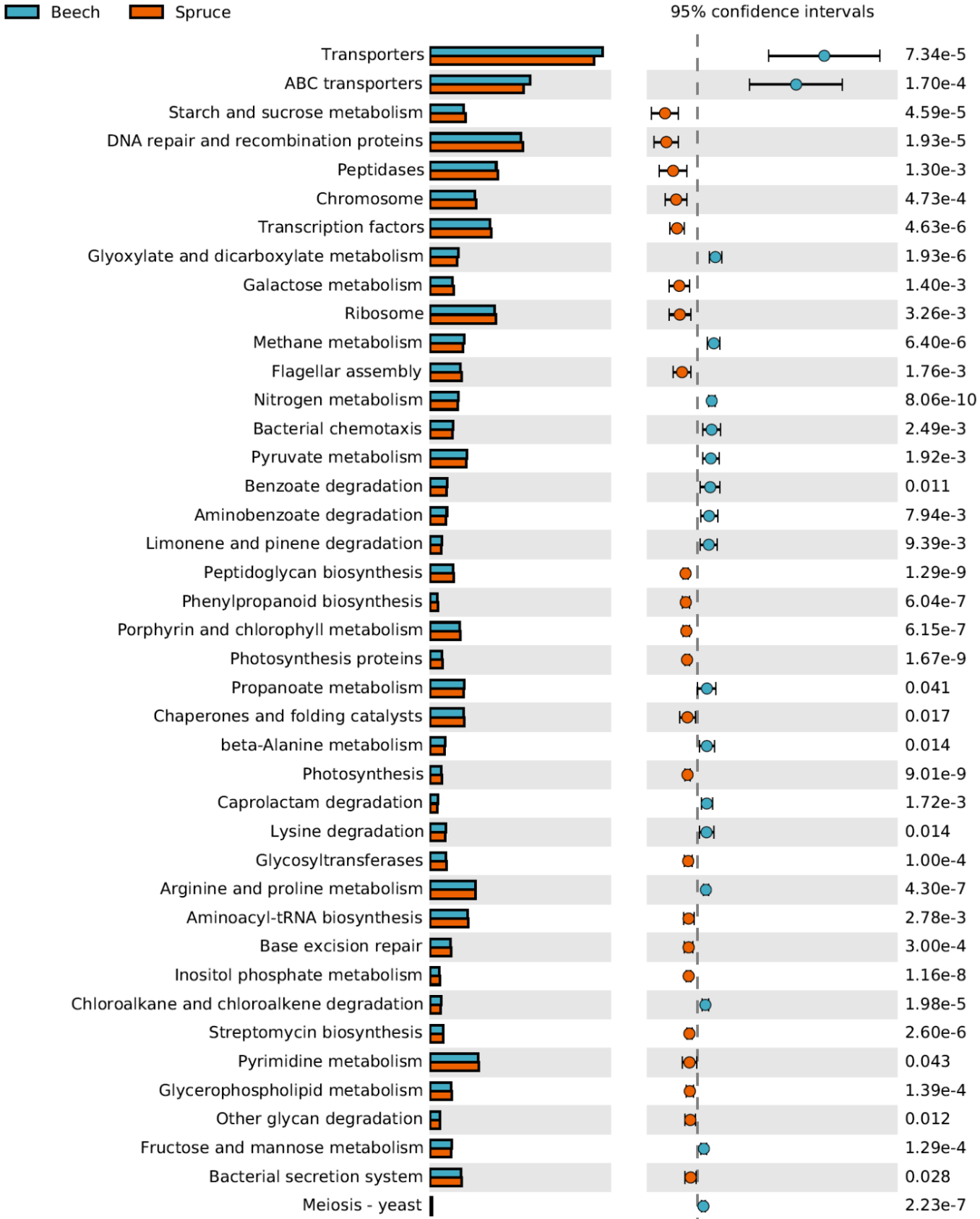


**Bárta *et. al*, Figure S4 (continue)**


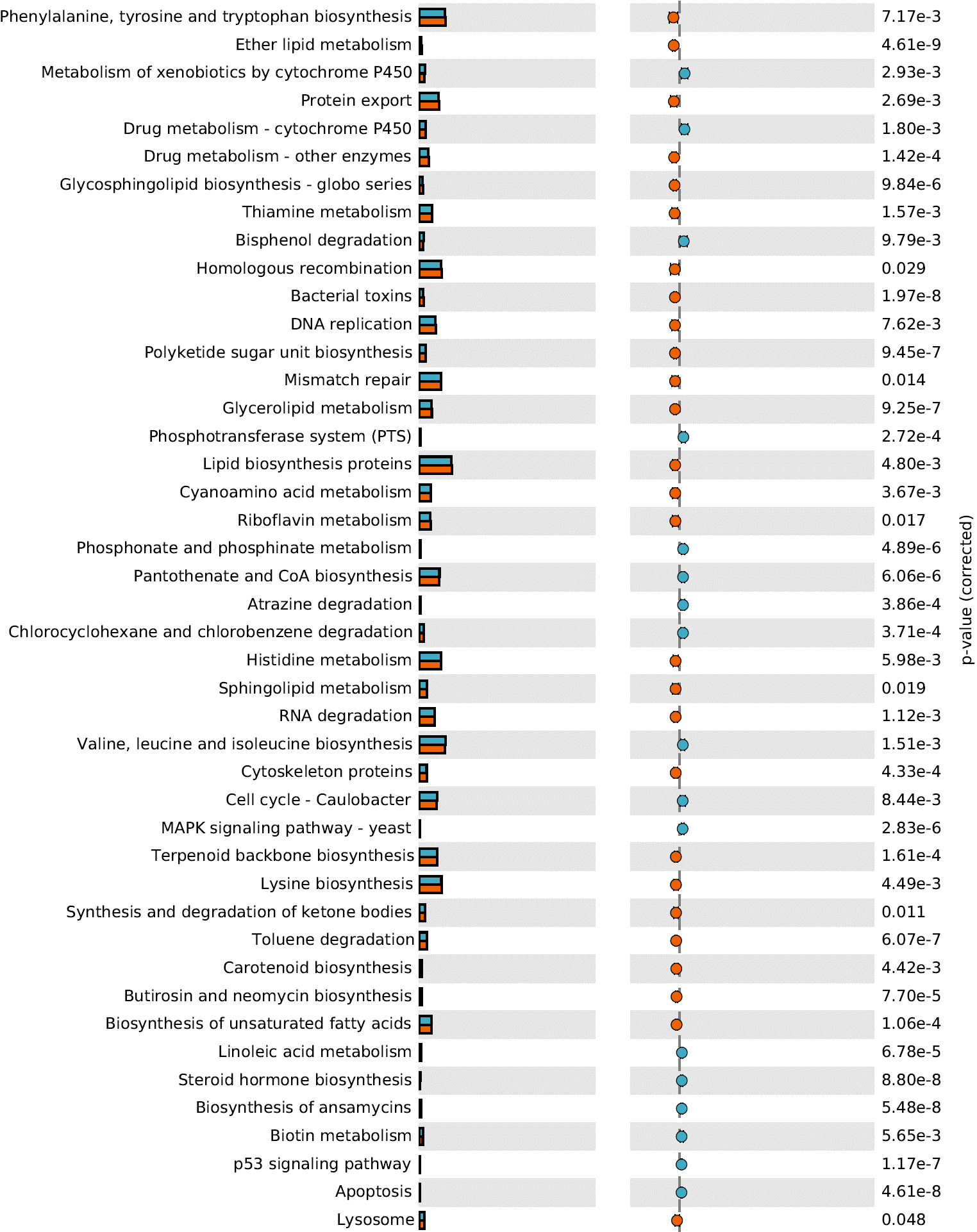


**Bárta *et. al*, Figure S4 (continue)**


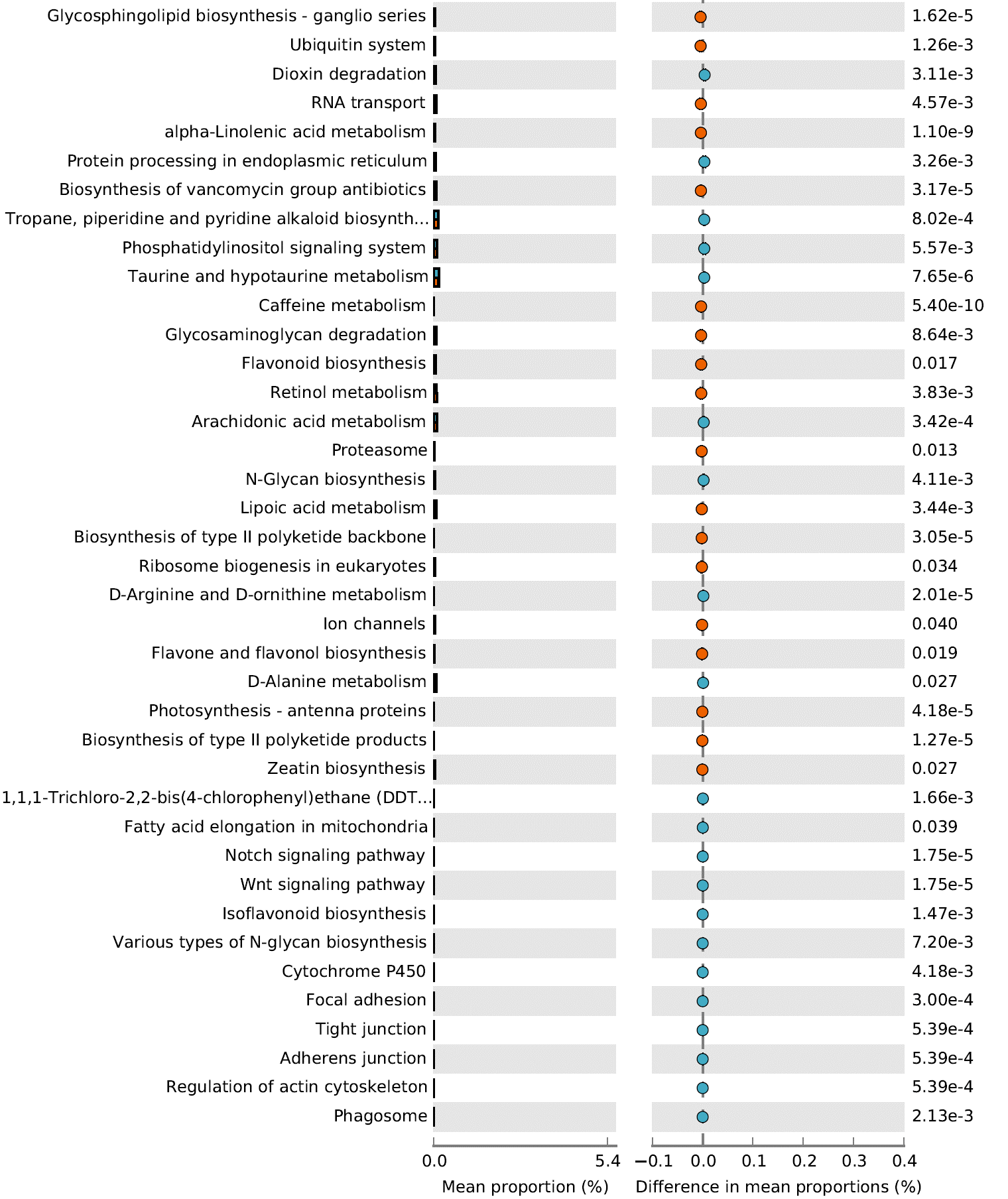


**Bárta *et al*., Figure S5**


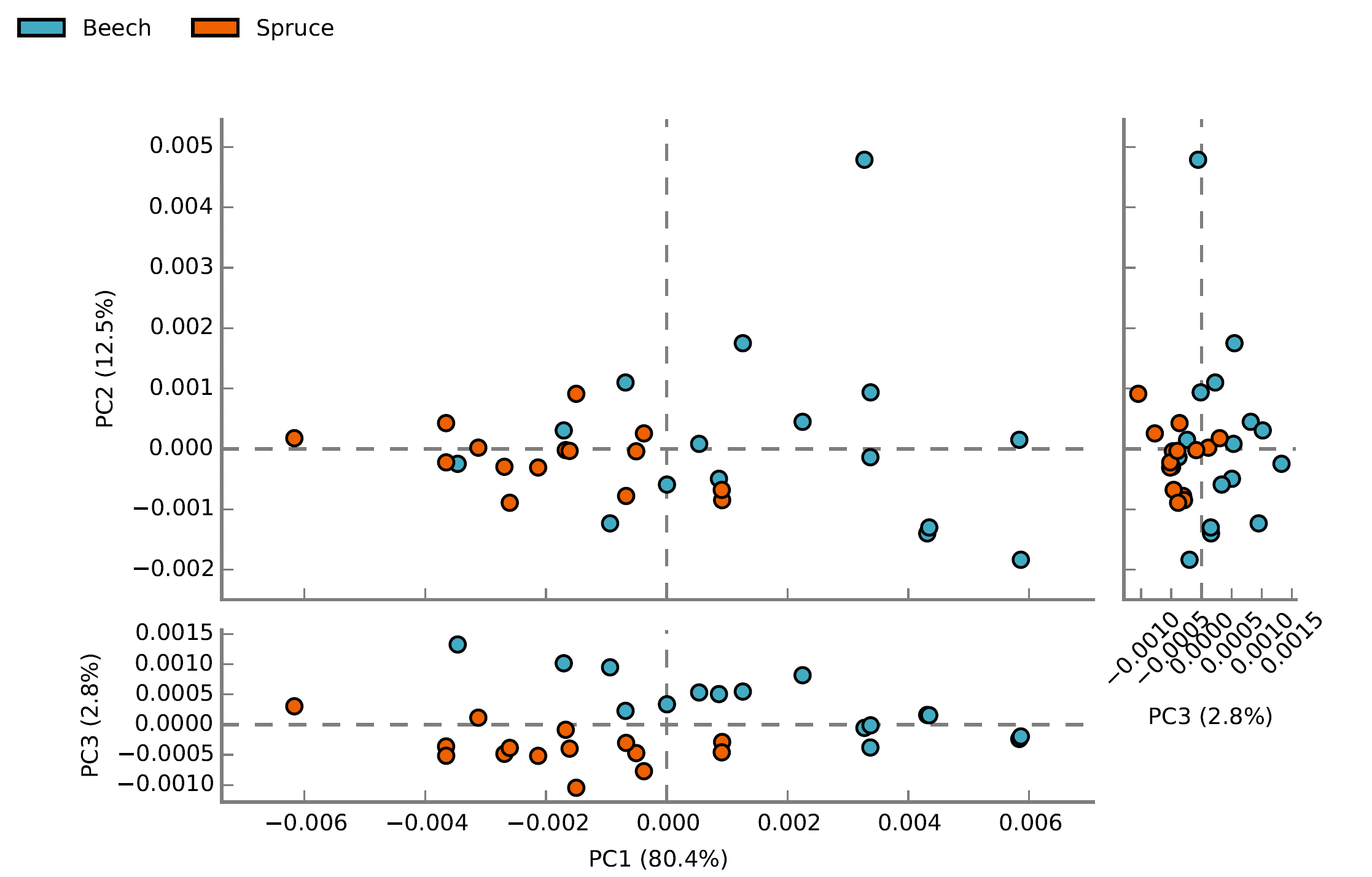


**Bárta *et al*., Figure S6**


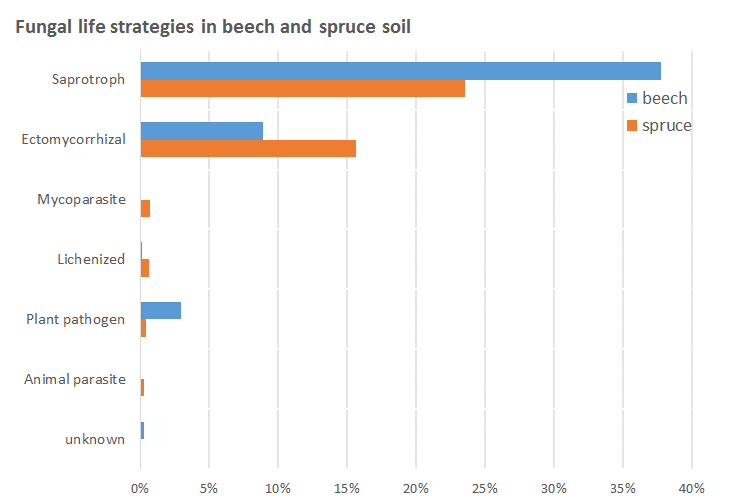


**Bárta *et al*., Figure S7**


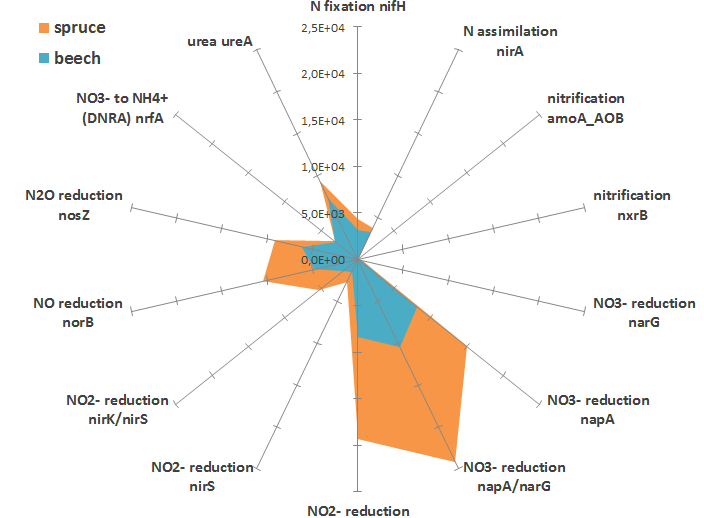


**Bárta *et al*., Figure S8**


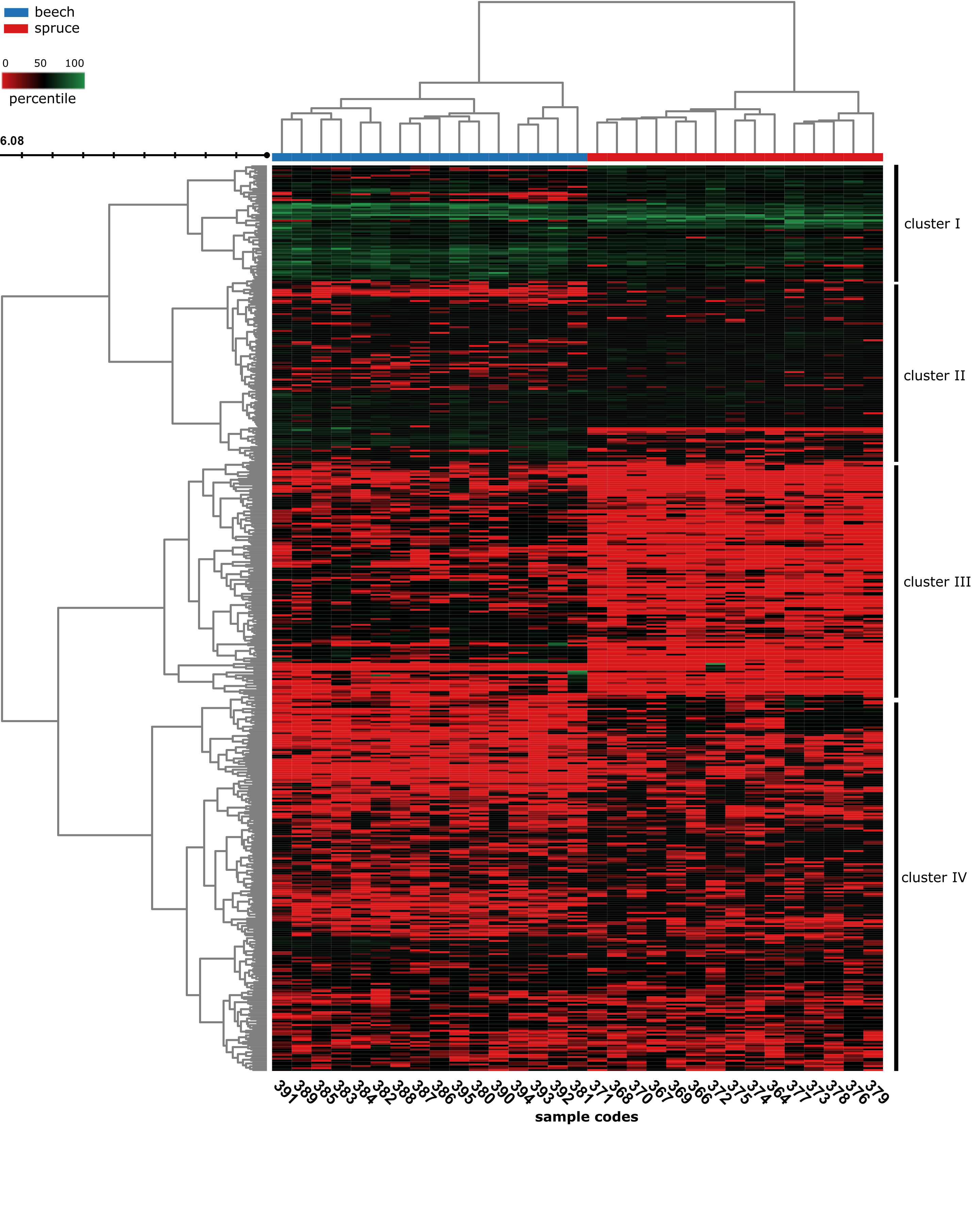


**Bárta *et al*., Figure S9**


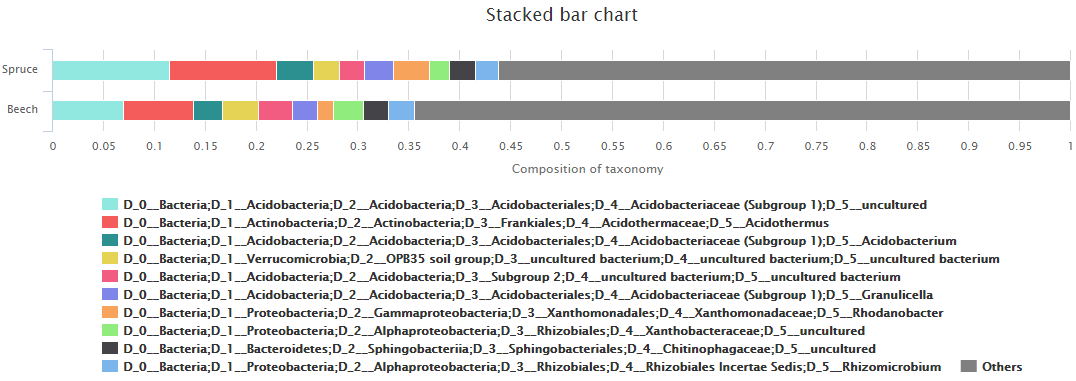


**Bárta et al., Figure S10**


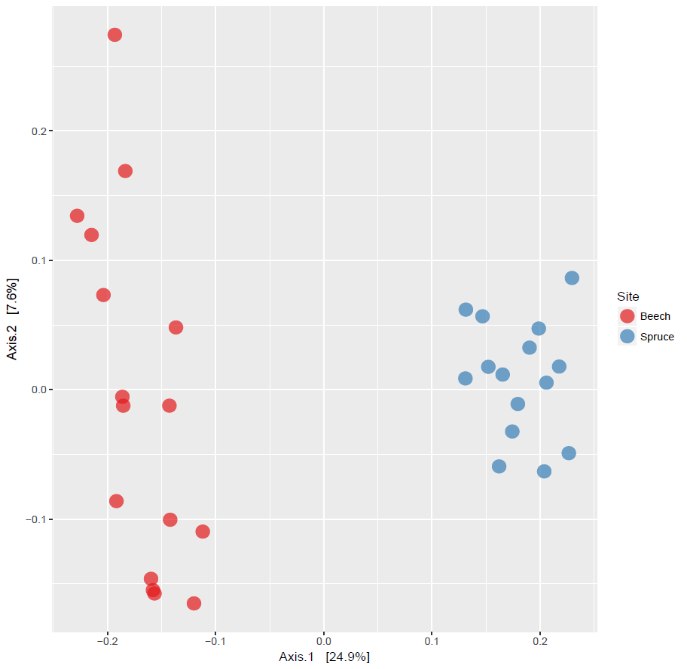

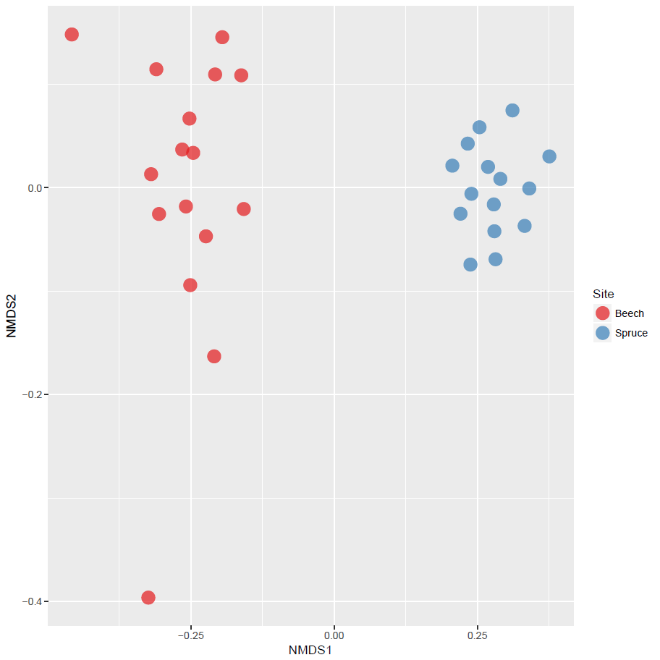


**b**

**a**

**Bárta et al., Figure S11**


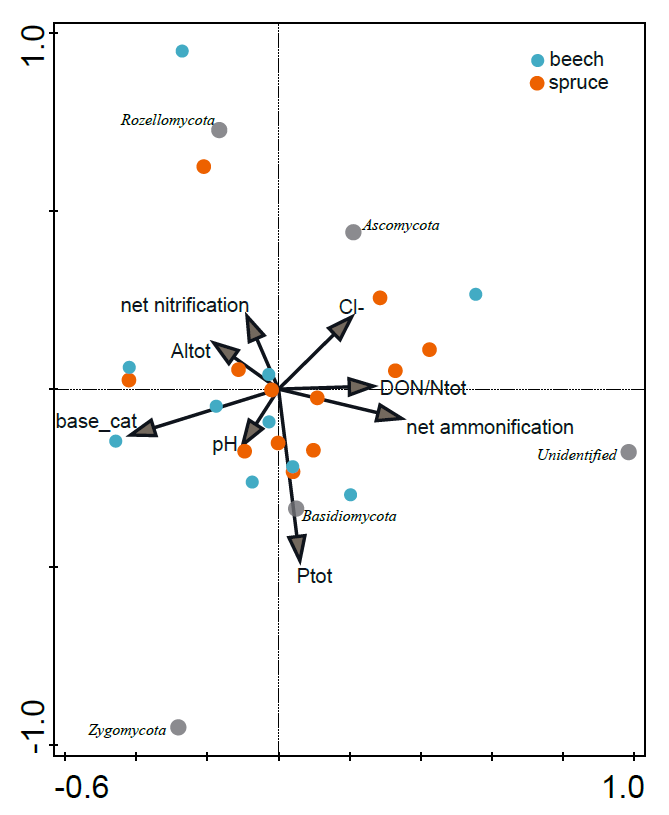


**Bárta et al., Figure S12**


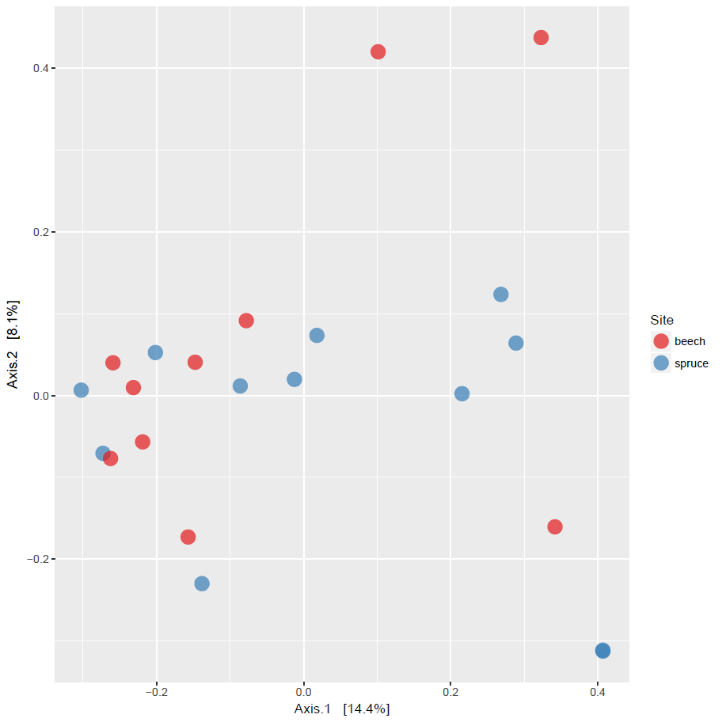


**a**


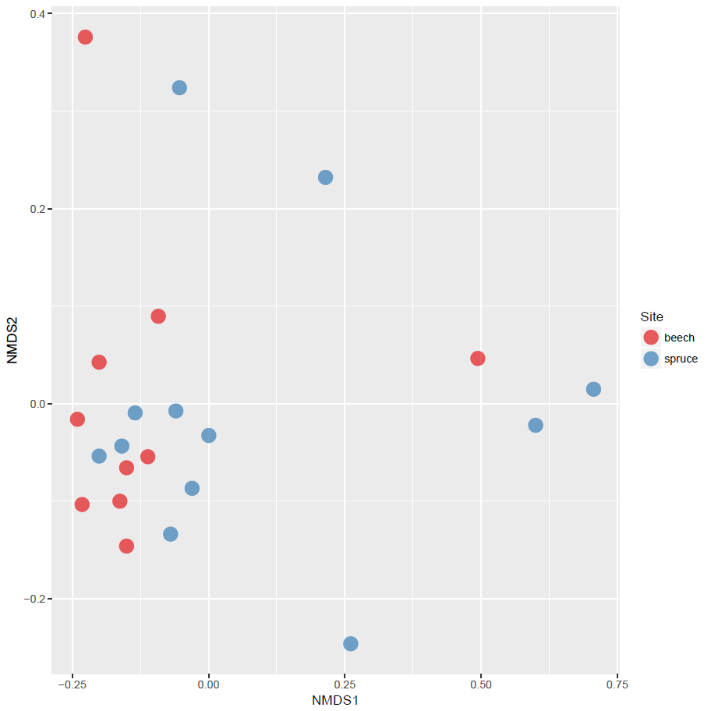


**b**

**Bárta et al., Figure S13**


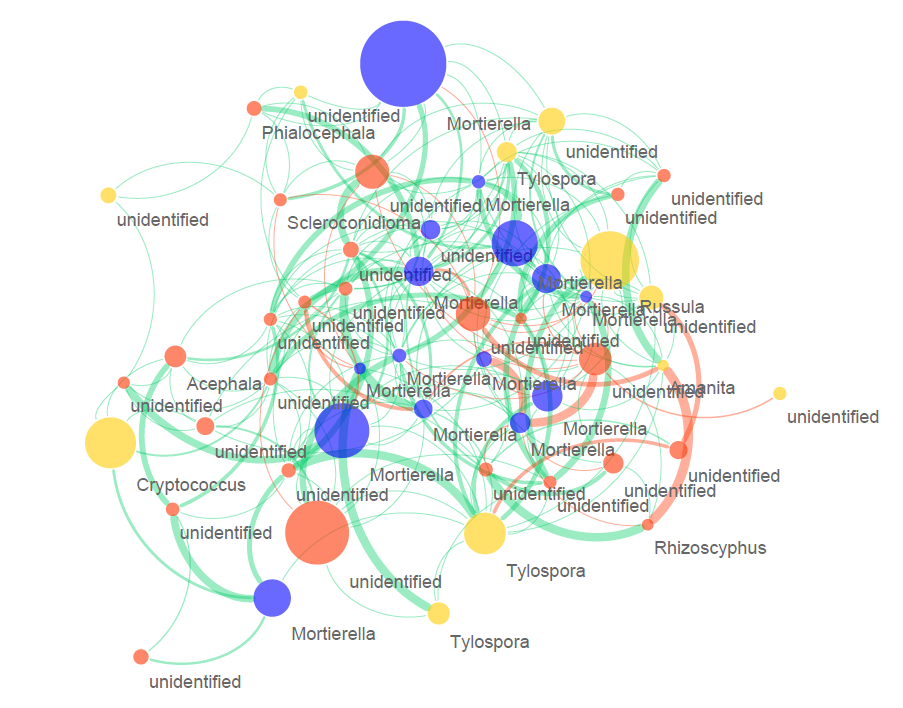


**a**


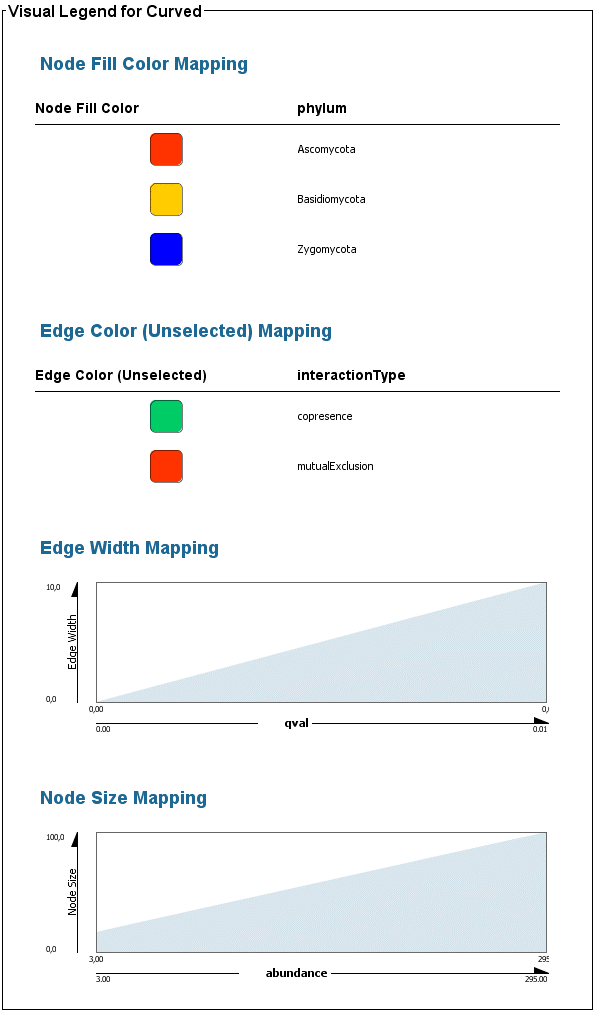

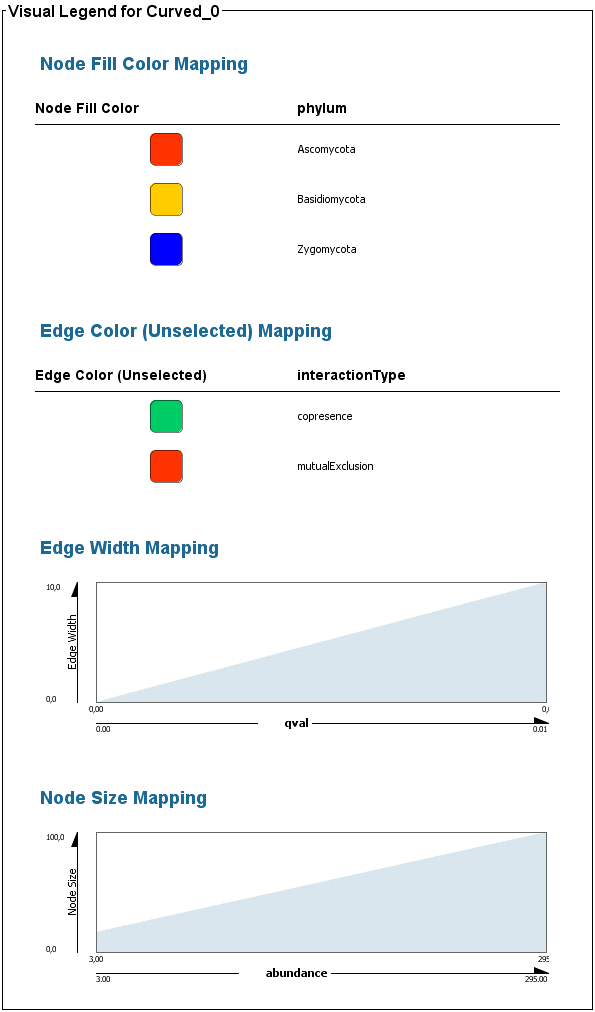

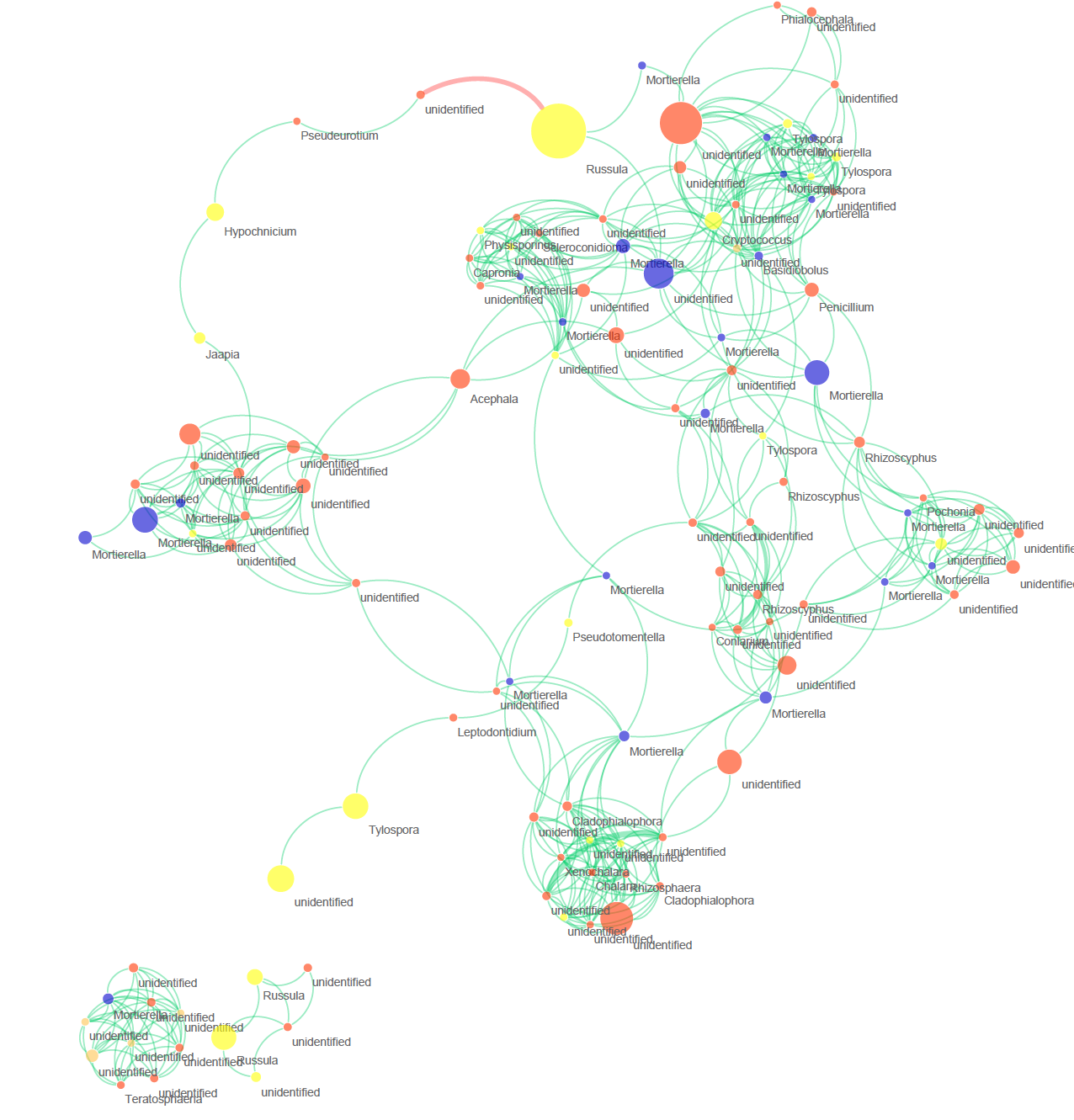


**b**

**Bárta et al., Figure S14**


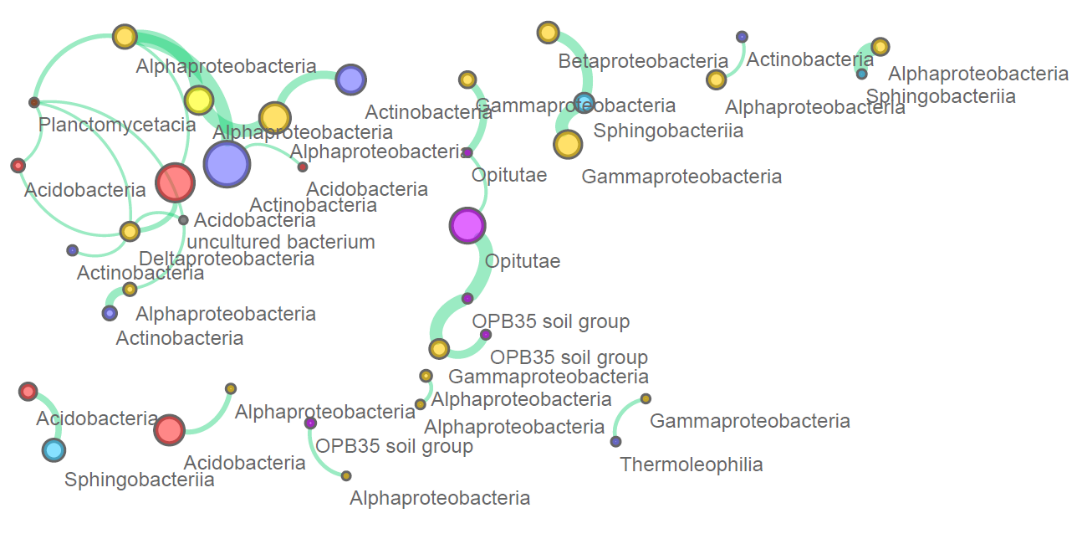

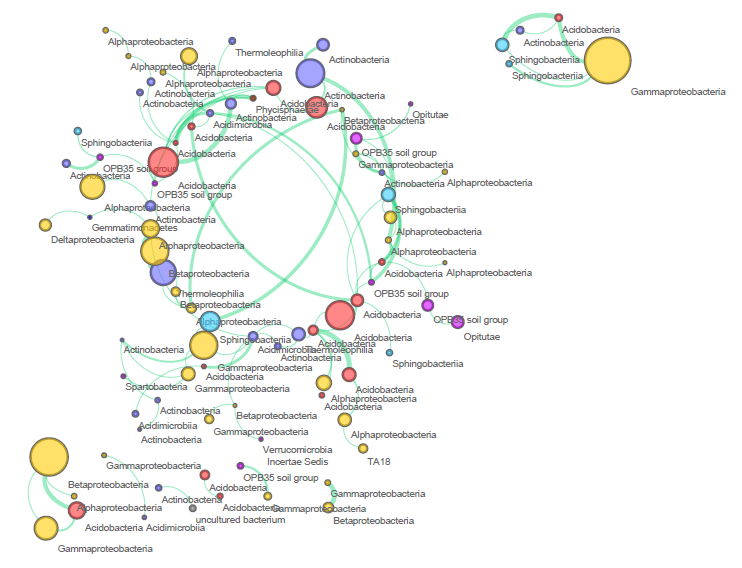


**b**

**a**

**Supplementary references**

Fish, J.A., Chai, B.L., Wang, Q., Sun, Y.N., Brown, C.T., Tiedje, J.M., Cole, J.R., 2013. FunGene: the functional gene pipeline and repository. Front Microbiol 4.

Oulehle, F., Evans, C.D., Hofmeister, J., Krejci, R., Tahovska, K., Persson, T., Cudlin, P., Hruska, J., 2011. Major changes in forest carbon and nitrogen cycling caused by declining sulphur deposition. Global Change Biol 17, 3115-3129.

Oulehle, F., Hofmeister, J., Hruska, J., 2007. Modeling of the long-term effect of tree species (Norway spruce and European beech) on soil acidification in the Ore Mountains. Ecol Model 204, 359-371.

Tedersoo, L., Bahram, M., Polme, S., Koljalg, U., Yorou, N.S., Wijesundera, R., Ruiz, L.V., Vasco-Palacios, A.M., Thu, P.Q., Suija, A., Smith, M.E., Sharp, C., Saluveer, E., Saitta, A., Rosas, M., Riit, T., Ratkowsky, D., Pritsch, K., Poldmaa, K., Piepenbring, M., Phosri, C., Peterson, M., Parts, K., Partel, K., Otsing, E., Nouhra, E., Njouonkou, A.L., Nilsson, R.H., Morgado, L.N., Mayor, J., May, T.W., Majuakim, L., Lodge, D.J., Lee, S.S., Larsson, K.H., Kohout, P., Hosaka, K., Hiiesalu, I., Henkel, T.W., Harend, H., Guo, L.D., Greslebin, A., Grelet, G., Geml, J., Gates, G., Dunstan, W., Dunk, C., Drenkhan, R., Dearnaley, J., De Kesel, A., Dang, T., Chen, X., Buegger, F., Brearley, F.Q., Bonito, G., Anslan, S., Abell, S., Abarenkov, K., 2014. Global diversity and geography of soil fungi. Science 346, 1078-+.
